# The Intestinal Microbiota May Be a Potential Theranostic Tool for Personalized Medicine

**DOI:** 10.3390/jpm12040523

**Published:** 2022-03-24

**Authors:** Marina Di Domenico, Andrea Ballini, Mariarosaria Boccellino, Salvatore Scacco, Roberto Lovero, Ioannis Alexandros Charitos, Luigi Santacroce

**Affiliations:** 1 Department of Precision Medicine, University of Campania “Luigi Vanvitelli”, 80138 Naples, Italy; marina.didomenico@unicampania.it (M.D.D.); mariarosaria.boccellino@unicampania.it (M.B.); 2 Department of Basic Medical Sciences, Neurosciences and Sensory Organs, University of Bari “Aldo Moro”, 70124 Bari, Italy; 3 AOU Policlinico Consorziale di Bari-Ospedale Giovanni XXIII, Clinical Pathology Unit, Policlinico University Hospital of Bari, 70124 Bari, Italy; robertolovero69@gmail.com; 4 Department of Emergency and Urgency, National Poisoning Centre, Riuniti University Hospital of Foggia, 71122 Foggia, Italy; alexanestesia@hotmail.com; 5 Department of Interdisciplinary Medicine, University of Bari “Aldo Moro”, 70124 Bari, Italy; luigi.santacroce@uniba.it

**Keywords:** microbiota, intestinal microbiota, oral microbiota, immune system and dysbiosis, probiotics, microbiota analysis, clinical microbiology, clinical biochemistry, laboratory medicine, theranostic and translational research

## Abstract

The human intestine is colonized by a huge number of microorganisms from the moment of birth. This set of microorganisms found throughout the human body, is called the microbiota; the microbiome indicates the totality of genes that the microbiota can express, i.e., its genetic heritage. Thus, microbiota participates in and influences the proper functioning of the organism. The microbiota is unique for each person; it differs in the types of microorganisms it contains, the number of each microorganism, and the ratio between them, but mainly it changes over time and under the influence of many factors. Therefore, the correct functioning of the human body depends not only on the expression of its genes but also on the expression of the genes of the microorganisms it coexists with. This fact makes clear the enormous interest of community science in studying the relationship of the human microbiota with human health and the incidence of disease. The microbiota is like a unique personalized “mold” for each person; it differs quantitatively and qualitatively for the microorganisms it contains together with the relationship between them, and it changes over time and under the influence of many factors. We are attempting to modulate the microbial components in the human intestinal microbiota over time to provide positive feedback on the health of the host, from intestinal diseases to cancer. These interventions to modulate the intestinal microbiota as well as to identify the relative microbiome (genetic analysis) can range from dietary (with adjuvant prebiotics or probiotics) to fecal transplantation. This article researches the recent advances in these strategies by exploring their advantages and limitations. Furthermore, we aim to understand the relationship between intestinal dysbiosis and pathologies, through the research of resident microbiota, that would allow the personalization of the therapeutic antibiotic strategy.

## 1. Introduction

The “*Human Microbiome Project*” aims to create reference bases for the sequences of microbial genetic material that exist in the human body, and to detect the relationship between the microbiota and humans, correlating the change in its composition with human health and disease. The natural organized microbial community, as well as their genes, found throughout the human body builds up the microbiota. More than 1014 microorganisms that make up the human microbiota have been identified and this number is likely to increase as it relates to the germs that have been discovered to date. Their genomes contain over than 2,500,000 genes [1]. Comparing the number of these genes with the human genome, the gut microbiome is considered the second genome as it contains 100 times more genes than the human microbiota; therefore, the microbiota that includes bacteria, ancient, protozoa, and fungi is unique for each person, and is linked to the types of microorganisms it contains over time and under the influence of many factors. However, studies do correlate several diseases with the gut microbiota as there are about 100 different species of pathogens that colonize the digestive system while the species discovered number more than 2000 [2,3]. Several studies have shown that microbiota can be associated with inflammation and disease such as urticaria, asthma, diabetes, obesity, irritable bowel syndrome (IBS), Alzheimer’s disease, central nervous system diseases, cancer, and others. The severity of these diseases has led not only to the research of the type of microorganisms present in the human intestine but also the modality of how it could induce a colonization by the “friendly” desired microorganisms to reduce or prevent such pathological situations [4,5,6,7]. 

## 2. Exploring the Intestinal Microbiota

The recent possibilities that various molecular biology techniques offer us have contributed to research for the more detailed study of human colonization of the intestine. The techniques focus on the rapid analysis of part of the bacterial genome and of the sequence of the small 16S subunit of ribosomal RNA [1,8,9]. 

In the 16S gene, in all species of bacteria and in the ancients, nine hypervariable regions are identified, symbolized as V1–V9, and containing 30 to 100 base pairs. Among these regions are conserved areas that can be exploited to design primers and sequence the gene. This sequencing procedure facilitates the classification of bacteria, with the most conserved areas being related to the highest classification, and the least conserved areas being related to genus and species. Today, 2172 species other than humans have been discovered, which have been classified into 12 different phyla [10,11]. Of these, 93.5% belong to *Actinomycetota Bacteroidota, Pseudomonadota*, and *Bacillota*. Of the 12 genera found, three phyla contain only one species isolated from humans; one of them has been isolated from the human intestine and is called *Akkermansia muciniphila* (the only representative of the genus *Verrumicrobia*) [1]. In addition, 386 obligatory anaerobic species have been identified in the human intestine but have also been found in areas of the mucosa such as the oral cavity, which is another meeting point for microbes from other parts of the organism [1]. Overall, the intestinal microbiota does not have a completely different microbial composition than other areas of the human body. These microorganisms contain a gene pool (microbiota) which has been estimated at around 106 genes so far. The identification of the genes showed a clear correlation with the geographic area in which the host resides. This discovery supports the view of the influence of environmental factors and the genetic background of the individual on the composition of the microorganisms that make up the human microbiota in general. The gut microbiota shows a wide variety of ancient and eukaryotic bacteria, a composition influenced by various factors. In the duodenum there are 10^5^–10^6^ bacteria, the final ileum has 10^8–^10^9^ (per gram of tissue or feces), and the large intestine has 1012 (per gram of intestinal tissue) with greater variety of bacteria than that of the tenuous intestine. The small intestine is rich in *Bacillota* and *Actinomycetota* phyla, while *Bacillota*, *Bacteroidota* phyla and *Lachnospiraceae* spp. are more numerous in the colon [12,13]. This bacterial difference is due to the acidic environment in the small intestine having a higher concentration of oxygen than in the large intestine [14,15].

## 3. Factors Affecting the Intestinal Microbiota 

### 3.1. Method of Delivery and Age

There is rising scientific literature regarding the fetus placenta, membranes, and amniotic fluid that claims the presence of microbiota. There is evidence for the “sterile womb” hypothesis which argues that the fetus is microbiota-free [16,17]. It seems the intestinal microbiota development begins immediately at human birth, even if samples from the uterine area are positive for the presence of microorganisms in the placenta. It appears that both vaginal and intestinal bacteria can access the fetus through different entry paths: the vagina with upward entry, and the intestinal by the dendritic cells of the immune system [18,19]. Recent studies show the presence of bacteria in placental tissue, umbilical cord blood, fetal membranes, and amniotic fluid from healthy newborns without signs of infection or inflammation. The meconium (first stools of newborns) of premature infants, born to healthy mothers, contains a specific microbiome, with the main phyla being *Bacillota* with a predominance of *Staphylococcus* spp., while *Pseudomonadota* phyla are found in species such as *Escherichia coli* (*E. coli*), *Klebsiella pneumoniae*, *Serratia marcescens* [6,17,20].

After birth, the gastrointestinal tract is enriched by various colonizing germs from both nourishment and the mother’s environment. This colonization can be disturbed or changed by disease, antibiotic treatment, changes in eating habits, etc. It has been shown that the composition is influenced by the birth process (natural or caesarean) [21,22]. Colonization occurs during natural birth by the inoculum, which generally consists of aerobic and possibly anaerobic bacteria (the newborn’s intestine initially contains oxygen), then is replaced by obligate anaerobic bacteria, which usually appear in the adult, and a welcoming environment is thus created [6,23]. Furthermore, there are a small number of different taxonomic categories with relative dominance of the phyla *Actinomycetota*, and *Pseudomonadota* which remain unchanged during the first month of life, but not in the following months, as there is a large increase in variability and new genetic variants. Maternal vaginal and fecal microbiota are the main sources of inoculation in babies born by normal delivery [21,23,24]. Indeed, newborns harbor microbial communities dominated by species of the genera *Lactobacillus* spp. (the most abundant genus of the vaginal and early intestinal microbiota), *Bifidobacterium*, *Prevotella* or *Sneathia* [6,24]. It appears that anaerobic microbes, such as members of the *Bacillota* and *Bacteroidota* phyla, which do not grow outside their host, rely on close contact between mother and infant for transmission. Finally, due to the presence of oxygen in the intestinal tract of the newborn, the transmission of severe anaerobes can occur not immediately at birth but at a later stage through the spores [16,25,26].

Newborns with a normal delivery show a high concentration of *Lactobacillus* spp. and *Prevotella* in the first few days that come from the mother’s colon area. In contrast, children born by caesarean section show colonization by the genera *Clostridium*, *Streptococcus, Propionibacterium*, and *Corynebacterium*. Indeed, the first bacteria observed in children born by caesarean section are those of the skin and the hospital environment and their intestinal microbiota is dominated by species of the genera *Corynebacterium*, *Staphylococcus*, and *Propionibacterium* but also with a lower number of populations and diversity of bacteria than children in the first weeks of life born through a normal birth [6,26,27]. Further evidence supporting the vertical transmission hypothesis is the similarity between the meconium microbiota and samples taken from potential infection sites. These “mother bacteria” do not remain indefinitely and are replaced by other populations during the first year of life [21,28]. Furthermore, this difference is also found in the microbiota analyses on feces of both mothers and infants with normal delivery showing that their microbiota is 72% like the microbiota found after microbial analysis of feces in the mother. On the other hand, the newborns by caesarean section showed a similarity of the microbiota of only 41% to that of the mother. Finally, the various objects around the newborn (animals, the mouth, the skin of the mother and relatives, and the mother’s milk) are secondary sources of inoculation of the microorganisms that gradually make up its microbiota. [6,21,22,28].

### 3.2. Breastfeeding and Eating Habits

During breastfeeding we see the growth of bacteria *Lactobacillus* and *Bifidobacterium* (such as *B. longum*, due to their ability to use specific oligosaccharides found in breast milk); their growth even exceeds that of the more common *Escherichia coli* and *Clostridium perfringens* bacteria [6,29]. These species degrade oligosaccharides and produce short-chain fatty acids, which cause the immune system to react and produce IgG immunoglobulins. In the early stages of development, the microbiota is made up of a small number of different microbes as its diversity is reduced with the colony of *Actinomycetota* and *Pseudomonadota* phyla [15,30]. During the first year of life, microbial diversity increases and has a great resemblance to the adult microbiota while it is characterized by a uniqueness for each child. Subsequently, *Enterococcus*, *Clostridium*, *Bacteroides*, *Enterobatteriacee* (such as *E. coli*), and *Streptococcus* spp. predominate in the composition of the microbiota. These first germs are linked to the immune system during the development of infants while its composition influences the degree of immune response [6,31,32]. Interestingly, human milk also plays a role in enhancing the production of IgA, cytokines, and cytotoxic lymphocytes by creating a strong defense mechanism. The microorganisms that grow can and metabolize insoluble carbohydrates. Such microorganisms are the microbes of the genera *Roseburia*, *Ruminococcus*, and *Eubacterium*. On the contrary, a nonvegetarian diet reduces the germs of the phyla *Bacillota* and increases those of the genus *Bacteroides* (*Bacteroidetes* phylum). Between two and three years old the complexity and functionality of the microorganisms that make up the microbiota is like that of an adult and always dependent on the personal host’s diet (Table 1) [33,34,35,36,37,38]. 

### 3.3. Living Conditions and Hygiene

The microbial composition of the gut is different in people living in developed countries than in people living in developing countries and in fact according to epidemiological data it is remarkable to observe the lower prevalence of allergic symptoms and asthma in developing countries. The explanation of the above phenomenon is supported through the hypothesis of hygiene in which excessive cleanliness leads to the reduction of infectious stimuli required by the immune system for its development [39,40]. The prevalence of larger families, higher residence in rural areas, worse conditions of hygiene and care, and less use of antibiotics in developing countries are conditions that support above case. Even differences in the diet of developed and developing societies lead to differences in the intestinal microbiota; e.g., in Japan, due to the high consumption of fermented rice products and fish, alongside good hygiene levels, we see low asthma incidence rates [5,41].

### 3.4. Individual Intestinal Secretory Function

There are factors that are produced by intestinal epithelial cells, namely the secretion of mucus, AMP (antimicrobial peptides), and IgA immunoglobulins, that help the growth of some species of microorganisms and inhibit the growth of others. Hence, they control the surface structure of the colon that is colonized, altering it and thereby affecting the composition of the microbiota [42,43]. In the large intestine, mucus plays a key role in blocking certain microorganisms from intestinal epithelial cells. Mucus consists of two layers, the inner one which does not contain microorganisms and the outer one that contains mucin, which has O-glucan, which provides a source of energy and adherence to the microorganisms of the intestinal microbiota [16,44]. The use of mucin in the growth of germs depends on two classes of enzymes, the hydrolases and lyases of the polysaccharides encoded by the genes of the microbes of the microbial flora. Some species in the gut microbiota (such as *Bacillus thetaiotaomicron*) can break down complex carbohydrates [45,46] since there are more than 260 genes in their genome that encode enzymes for the cleavage of such molecules. Intestinal germs can also modify the amount of mucus produced by intestinal mucosal cells. Mucus could reduce infection by pathogens directly by attaching to them, protecting epithelial cells from acidic and enzyme-secreted secretions, and even being the means by which products of bacterial metabolism are collected and activate the body’s defenses. It is therefore clear that the interaction of a microorganism with its host is complex and is influenced by many of the factors mentioned above [47,48]. Under suitable conditions there may exist a long-term symbiosis with many benefits for the host’s health. The mucus in the small intestine is not abundant and the main role for the creation or modification of the gut microbiota is played by the AMPs. They are induced by Paneth cells through a mechanism in which PRR (pattern recognition receptors) are involved, and which are activated by various microbial components (such as lipopolysaccharides) through a pathway called microbe-associated molecular patterns (MAMP) [49,50]. The PRR–MAMP system promotes the action of the mucus barrier by inducing the production of IgA immunoglobulins, mucus, and AMPs. The concentration of AMP is higher in the crypts of the intestinal epithelium since there are Paneth cells. Secreted AMPs are the first line of defense against the presence of bacteria, viruses, fungi, and tumors that cause the secretion of various proteins, such as the Reg proteins, various ribonucleases, etc. [51,52]. Some species are resistant to high concentrations of AMP such as the genus *Bacteroides*, more common in the microbiota. Furthermore, antibacterial lectins form pores in the membranes of Gram-positive bacteria, thus inhibiting their approach to the intestinal mucosa. Plasma cells present in the intestinal mucosa produce the immunoglobulin IgA, with have the action controlling bacteria growth locally, and may additionally bind to specific receptors on the bacterial membrane, preventing biofilm formation [53,54,55]. 

### 3.5. The miRNAs

The miRNAs are small fragments of RNA with a length of 18–23 nucleotides that do not encode genetic information and are generated in the nucleus. They are transferred to the cytoplasm by silencing genes by binding to the untranslated 3 region, accelerating mRNA destruction, or inhibiting translation [56]. Only miRNA can regulate miRNA α, and have been found to be extracellular so, in this way can circulate in body fluids. MiRNAs have also been found in human feces and their type appears to be associated with the onset of malignant tumors. Intestinal epithelial cells and Hopx positive cells are the main sources of miRNA. Thus, these miRNA fragments are released by the host’s intestinal cells, enter intestinal bacteria, and regulate the transcription of genes, thus influencing bacterial growth. In a study on animals (mice), the presence of miRNA from the host’s intestine was found in the feces of those used for experimentation. In some mice the DNA was modified to block the synthesis of miRNAs and in which the germs of the microbiota grew in an uncontrolled way. This group of mice exhibited intestinal disturbances (colitis and other) but if miRNA molecules were administered, the growth of the bacteria was stimulated and the disturbances diminished [57,58,59,60]. The inability of intestinal epithelial cells to produce miRNA has been associated with colonization of the gut with microbes negative to human health. In addition, the intestinal miRNAs from intestinal epithelial cells or external diets interact with intestinal microbes and modulate their composition and distribution of the intestinal microbial ecosystem. MiRNAs can thus also regulate the intestinal immune system by influencing the innate immune system via regulation of NOD2 and TLR, two of the crucial PAMPs. Additionally, they facilitate the differentiation of Th1, Th2, Th17, or Treg cells, also influencing the adaptive immunity cells. Thus, the human organism influences the composition of the bacterial population it hosts with this mechanism [57,58,59]. Various miRNAs enter bacterial cells and cause them to grow through gene expression, e.g., miRNA515-5p promotes the growth of *Fusobacterium nucleatum*, which has been involved in colorectal carcinogenesis, while miRNA-1226-5p causes the development of *Escherichia coli*. This suggests several potential therapies for the microbiota alterations (quantitative and qualitative) and chronic gut inflammation; hence, new research focuses on the possibility of using miRNA fragments for intestinal dysbiosis in the treatment of intestinal diseases (such as IBD, colon/rectal cancer, and others) [59,60,61,62].

### 3.6. Antibiotics

The use of antibiotics has a double effect on the organism host. The reason they are put into therapy is for the destruction of pathogenic microorganisms. However, in addition to this, the useful microorganisms of the intestinal microbiota are also destroyed, leading to the disappearance of some useful microbes and the change of the microbiota. Antibiotics interrupt the competition between pathogenic and nonpathogenic microbes, which leads to the restriction of nonpathogenic microbes [63]. Disorganization leads to the growth of pathogens, such as *Clostridioides difficile* bacteria. *C. difficile* is Gram positive (mandatory anaerobic), is found in the intestine in approximately 5% of the adult population, and causes pseudomembranous colitis in people treated with antibiotics, thus causing a microbiota dysbiosis that allows *C. difficile* to overgrow in individuals who have already colonized, while making the individual more vulnerable to the settlement of *C. difficile* spores of exogenous origin [64,65]. Furthermore, it was noted that this effect on the microbiota does not appear to be transient; there are antibiotics, such as clindamycin, which cause changes by inhibiting the growth of some microorganisms even two years after their intake. Similarly, clarithromycin (used to treat *Helicobacter pylori*) reduces the *Actinomycetota* phyla, while ciprofloxacin reduces *Ruminococcus* [66,67,68,69]. Vancomycin, which is considered the best therapeutic approach for *C. difficile*, causes a change leading to the development of pathogenic strains of *Escherichia coli*. Finally, the use of antibiotics in farms such as poultry and cattle in small doses increases their growth and weight, which is particularly important for the economy of meat production. It has been noted that this could be the cause of obesity in humans and appears to be associated with changes in the gut microbiota that is involved in this pathology [68,69,70,71,72,73].

## 4. Intestinal Microbiota Modulation

### 4.1. Probiotics

Probiotics are microorganisms that, when taken in appropriate doses, protect human health. The most common probiotics are species of *Lactobacillus*, *Bifidobaceria*, and yeasts, such as *Saccharomyces boulardii*. One mechanism by which probiotics contribute positively to human health is the promotion of the growth of beneficial microbes in the intestinal microbiota [74,75,76]. Probiotics compete with pathogenic microbes in the intestinal tract, e.g., some *E. coli* spp. move and attack pathogenic microbes protecting the human microorganism. Some probiotics can also produce antimicrobials that kill pathogens. Indeed, the production of the substance reuterin by *Lactobacillus reuteri* induces the immune response of the human body. Studies have shown that the use of probiotics and the change in the microbiota that it causes contributes positively to various pathogenic conditions [77,78,79,80]. Chronic periodontitis, urinary tract infections, necrotic enterocolitis, and treatment of elevated total cholesterol levels appear to be positively associated with the intake of probiotics, and therefore with the microbiota. Taking probiotics seems to contribute positively to the better health of people with diabetes as in people with type 2 diabetes there was a reduction in blood glucose and HbA1 levels. Finally, probiotics could protect microbiota from environmental factors, depending on their dose. For example, the use of larger amounts of probiotics appears to have a positive effect on the cessation of diarrhea by exogenous factors [81,82,83,84]. 

### 4.2. Prebiotics

Prebiotics are substances and ingredients that are usually produced by microorganisms, while their intake helps maintain and grow the beneficial microorganisms of the intestinal microbiota. Prebiotics usually consist of indigestible carbohydrates, oligosaccharides, small polysaccharides (such as inulin), fructose, lactofructose, etc. [6]. Prebiotics should be gastric acid resistant but can be metabolized by enzymes, to be absorbed by the digestive system and used by intestinal microbes. Prebiotics affect various species of microbiota’s bacteria found in the colon, with the main target being bacteria of the genera *Lactobacilus* spp. and *Bifidobacteria* spp. [85,86]. The consumption of fiber is very important in its creation of mucus that acts as a barrier. Various studies have shown the effect of prebiotic fibers in the formation of the intestinal microbiota, e.g., taking inulin can protect against the negative effects of a high-fat diet [87,88,89]. Finally, other facts indicate that the intake of a small amount of fiber typically contained in a Western diet causes the reduction of protective mucus, leading to microbiota changes, which result in the creation of inflammation and other pathological conditions. Thus, the low-fiber content is associated with the appearance of chronic diseases [89,90,91].

### 4.3. Postbiotics 

The definition of postbiotics from the ISAPP (International Scientific Association for Probiotics and Prebiotics) is: “… *a preparation of inanimate microorganisms and/or their components that confer health benefits to the host…*”. Postbiotics are essentially microbial cells or their deliberately inactivated cellular components, with or without metabolites, which confer a health benefit [92,93]. Hence, postbiotics are the metabolically active products of probiotics. An emerging approach in microbiota enhancement is to first identify the molecules that are missing in a particular disease, and then supplement the diet with the missing molecules or precursors that can be converted into bioactive molecules by the microbial population. This approach is important given that postbiotics are an important class of functional molecules used by the microbiota to modulate human health. Amino acid derivatives transformed by the gut microbiota are part of a class of compounds that are potentially postbiotic [94]. For example, indole, which may be derived from tryptophan, reduces inflammatory mediators, transcription of proinflammatory factors, and colonization of intestinal epithelial cells by pathogens, while increasing tight-junction resistance and the production of mucin. The changes in the amount of butyrate, acetate, and propionate have also been correlated with the deterioration of health in older people, which is further evidence of the importance of bacterial production of SCFAs in the physiology of the gastrointestinal tract [93,95]. 

### 4.4. Parabiotics

Another category with functional ingredients, as scientific evidence has shown, is the integration with inactivated microorganisms, the so-called paraprobiotics, which can provide health benefits. The term paraprobiotic defines inactivated probiotics, that is, “nonviable” microbial cells (intact or in fractions) or crude cell extracts (i.e., only the complex chemical composition), which, if administered (orally or topically) in adequate quantities, can confer a benefit to the person and also to the animal [92,96]. This inactivation of these microorganisms (bacterial cells) can be obtained through physical or chemical treatments, such as heat treatment (which seems to be the most effective because it better preserves the structure of the cell components), UV rays, specific enzymes, or mechanical treatments, or by pressurization, freeze drying, or acid deactivation [92]. The application of a specific inactivation process for each strain is undoubtedly the optimal method. A particular method of inactivation would be tindalization, which must be adapted for each of the selected strains. Tindalization is a delicate heat treatment capable of preserving the molecular membrane and cellular structure of microorganisms by inhibiting their ability to reproduce. The production of microbiologically nonviable but functionally active cells is guaranteed, which are stable and still able to positively influence human and animal health [96,97].

### 4.5. Fecal Transplantation

Fecal transplantation is a transfer process of the fecal microbiota from a healthy donor to a patient with some bowel diseases, or to a person whose microbiota has changed due to various factors. The mode of transport can vary; the most appropriate is still a field of research and is related to the specific disease to be treated [98]. The methods that have been studied for the transfer of the microbiota are the colonoscopy, the enema, the rectal catheter, capsules that contain lyophilized bacteria, etc., with greater efficiency for observation during a colonoscopy and enema [99]. The transfer leads to changes in the recipient microbiota for the individual to accept the beneficial effects of the germs present in the donor. The first stool microbiota transplantation took place in the 4th century in China to combat food poisoning and control diarrhea. In modern medicine it is asserted that the change and enhancement of the microbiota of patients with “foreign” microbiota is good practice in the treatment of pseudomembranous colitis. Today, the technique is used to treat colitis from *C. difficile*. The treatment rates are close to 90%. In addition, the method has been applied to the treatment of irritable bowel syndrome (IBS), autoimmune diseases, metabolic diseases, etc. [100,101,102,103]. The value of the intestinal microbiota is enhanced by the observation that showed that the results in treatment are influenced by the donor microbiota. Thus, there are donors who are referred to as “super donors”, the characteristics of whom are fully described and include great microbial diversity with a predominance of germs that have beneficial effects on the individual. Additionally, the donor’s suitability varies depending on the disease for which they are selected [104]. 

## 5. Methods of Microbiota Analysis

### 5.1. Culture in Batch and in Continue

The culture in batch consists of the simple incubating samples or single strains of a species of interest in a complete culture medium, i.e., containing all the nutrients for that bacterium. Then, the single bacterial colonies are isolated to describe their phenotypic characteristics and their metabolic capacities. Thanks to this type of approach, it has been possible to cultivate over 1000 distinct bacterial species, isolated from the human gastrointestinal tract alone [105,106]. They make it possible to compare bacterial groups of interest, based on their growth rates and the production of metabolites on different substrates or on the species-specific interactions that are formed. Batch cultures obviously have limitations for two main reasons [107]. The first is that the results are obtainable only for short periods of time, due to the exhaustion of the nutrients present in the culture medium or the formation of toxic substances for the bacterial species of interest, which limits their growth. Secondly, the preparation of a bacterial culture can be very expensive, since it may be necessary to make many different culture media, to recover as many bacterial species as possible within a sample [108,109,110]. The culture in continue is a method that consists in the use of open or continuous defined systems such as fermenters and drainage systems. With the fermenter it is possible to introduce in a stable and continuous way the growth factors and nutrients, removing with the drainage, dead cells and toxic substances produced by bacteria. This type of system is commonly used to study the characteristic microbiota of the colon because it is possible to perform cultures with sequential and distinct growth phases to reproduce the many environmental changes that microorganisms undergo in transit within it [111,112,113]. 

### 5.2. Animal Models Procedure 

The bacterial species of interest can also be cultured and maintained in animal models. The use of the animal model germ-free mice (which are animals completely free of microorganisms both internally and in the skin) are particularly useful for the study of microbiota bacteria. In fact, they can be easily inoculated with strains of interest and allow us to study the bacterium–host and bacterium–bacterium relationships in a simple and intuitive way. In contrast, knockout mice are genetically modified animals used to evaluate the effects of suppressing the expression of a certain gene [114,115,116]. 

### 5.3. Sequencing-Based Methods 

The most frequently used marker genes in ribosomal RNA sequencing are those contained in the RNA of the minor ribosomal subunits (called 16S and 18S) in bacteria and archaea, and in eukaryotes, respectively. There are gene sequences that are remarkably conserved in the different bacterial species and/or are present from a certain bacterial group or genus, but they are variable [117,118]. Therefore, this biomolecular test uses these regions that, after the extraction of the genetic material from the sample, amplify the marker genes by the polymerase chain reaction (or PCR) method using primer sequences, which is very specific for highly conserved regions, over time. Finally, a mixed group of amplified extractions is created which are derived from the largest possible number of bacterial species included in the biopsy sample and which are subsequently sequenced [118]. The obtained data are then grouped into operational taxonomic units (OTUs), organized in different clusters of sequence similarity, and should reflect, with reasonable approximation, the heterogeneous set of bacterial groups included in the sample. Ribosomal RNA sequencing can also be used to analyze fewer common genes within the microbiota since they are not ubiquitous and expressed only by some of the bacterial species contained in it, e.g., the genes expressed by microorganisms present in the colon that produce butyrate and propionate [119]. Given the great efficiency of the latest generation sequencing technology, it is possible to carry out the simultaneous sequencing of many bacterial genomes in the same session. This is thanks to the Multiplex PCR technique (a variant of PCR), which allows you to quickly identify deletions and/or duplications in a large gene [120]. Indeed, Multiplex PCR uses multiple primer sets in a single reaction mix and thus analyzes multiple genes in a single PCR run. A limitation of the complete genome sequencing method is that we need to have enough DNA for subsequent analyses. In 1995, *Haemophilus influenzae* was the first genome completely sequenced using the chain-termination sequencing (Sanger method) [121,122]. To achieve this, the bacteria of interest must first be cultured and most of the bacterial species present in the microbiota have not yet been cultured in the laboratory [123]. 

Metagenomics is a method that enables us to derive genomic sequences of interest from the extracted DNA from an environmental sample. Subsequently these are compared bioinformatically together with as many known sequences expressed by single bacterial species, excluding those that are not relevant for the study. Metagenomics is a technique by which it is possible to accurately determine the functional capacity of a bacterial community of interest. In some cases, however, the complexity of intestinal bacterial habitats (e.g., the colon) requires a greater sequencing effort to obtain sufficient data to compose a representative picture of the microbes present within them [124]. Single cell sequencing (SCG) is a complementary method to metagenomics and consists of the isolation of single bacterial cells from samples and the subsequent amplification of their entire genome to know what specific functions it is able to express. This type of method is combined with cell selection techniques, such as fluorescent in situ hybridization (FISH) and/or labeling with stable isotopes, which allow the recovery of bacterial cells that derive from a specific phylogenetic profile or that perform a particular function [125,126,127].

Finally, metatranscriptomics is the study of the transcripts of a bacterial community, which aims to identify the functions performed by the microorganisms that compose it, at a given time and in certain environmental conditions, unlike metagenomics which establishes its functional potential as absolute value [128]. This technique consists first in the isolation of RNA from an environmental sample and its subsequent use for the creation of retro-transcribed cDNA “libraries”. Therefore, it has both the creation of cDNA and the elimination of the host and bacterial ribosomal RNAs from the sample of interest, where they constitute the most represented class of RNA. An important limitation of metatranscriptomics is that due to the short half-life of the messenger RNA (which can be measured in a few minutes and thus the results obtained may not be representative of the bacterial activities carried out in situ) [129,130]. 

### 5.4. Molecular Methods 

The DNA fingerprinting method allows us to obtain useful universal genetic markers of bacterial genomes and therefore of the species present in a given particular microhabitat, such as the colon. First, the sample of interest is extracted and the DNA purified. Subsequently this DNA is divided into fragments by endonucleases. These enzymes perform divisions at certain nucleotide sequences, which are specific to each enzyme. These resulting restriction fragments are then separated by length by agarose gel electrophoresis. Subsequently, through the Southern blot hybridization technique, the bands generated by hybridization with radioactively labeled probes of known sequence or through fluorochromes are found. The differences between the genotypes are highlighted by the number of bands that appear using the same probe for hybridization, which is in turn determined by the number of cleavage sites present in the sequence considered [131,132].

Another method is the DNA microarray that consists of a network of DNA probes attached by the inverse hybridization technique to a solid support (such as glass, plastic, or silicon). Thus, the network marks the nucleic acid to be identified and allows us to simultaneously check the RNAs produced by thousands of genes and to evaluate the variations of their expression. The phylogenetic microarrays essentially consist of an array containing short oligonucleotides (the target of which is usually represented by the RNAs of the minor ribosomal subunit), which are selected to include the taxonomic range of microorganisms that are assumed to be present in each sample environmental [133]. DNA is extracted from the sample and ribosomal RNAs are amplified and labeled with a fluorochrome and finally hybridized against the microarray. Therefore, when the DNA spots of the microarray show a fluorescent signal, it will be confirmed that the taxonomic range chosen includes that of the bacterial species of the sample. An important limitation is that a small number of bacteria can be detected, i.e., those whose taxonomic range is included in the probes attached to the microarray. However, arrays are available including the complete taxonomic range of bacterial species present in niche microhabitats, associated with the human organism, such as the intestine or the oral cavity, etc. [134,135]. 

### 5.5. Quantitative Methods

The quantitative PCR (or real-time PCR) is based on the measurement of the fluorescence emitted by a DNA of interest during amplification by PCR: the amount of signal generated and the rate at which this signal accumulates, as the number of PCR cycles, allows you to measure the amount of target DNA present in the sample. This technique is often used to quantify the total number of bacterial cells contained in a sample. Furthermore, it can allow the quantification of the populations present in different bacterial groups, using a series of specific primers. A limitation of real-time PCR is that it allows only bacterial groups to be monitored for which specific primers have been constructed and therefore, the excluded groups will not be quantified, unless multiple primer sets are used [136,137].

The fluorescent in situ hybridization technique (FISH) requires the bacterial cells in question to be first fixed using chemical agents (e.g., formaldehyde) and then become permeable, to allow access to the fluorescent oligonucleotide probes. These oligonucleotides are approximately 15–30 bp in length and are commonly created to identify ribosomal RNA regions of selected phylogenetic groups [138]. The probes hybridize to any complementary rRNA sequence and display cells that have shown a positive signal. FISH, in addition to being a quantitative approach, has the advantage of allowing the observation of cells of interest in situ, with which it is possible to define a specific composition of bacterial groups present on the mucous membranes or skin. A limitation of this method, which is less sensitive from a quantitative point of view, compared to real-time PCR (qPCR), due to the rich presence of bacterial populations [139,140]. 

### 5.6. Functional Methods

Metaproteomics is the study of the set of peptides/proteins produced by mixed bacterial communities. Therefore, it provides functional information on them, allowing you to note all the changes in the expression of proteins within the microbiota, in response to changes in normal environmental conditions. This approach requires the proteins to be first extracted from the environmental sample of interest and then separated for their characterization by means of mass spectrometry so we can proceed with the comparison of the reference bioinformatics data present in the main databases [141]. The proteins/peptides are separated by liquid chromatography. Metaproteomics offers significant advantages on the studies of the gene expression of microbes within natural environments. This is because targeting proteins rather than messenger RNAs provides an extended and representative view of the functional activities carried out by the microbiota, also offering an explanation to the post-translational modification processes. Furthermore, proteins/peptides are also commonly more stable than messenger RNAs and from this it follows that the results achieved are no longer conditioned by the speed at which the samples are processed. This method has certain limitations, e.g., only proteins produced by the most representative members of the microbiota can be recovered to a reasonable extent [142,143]. Metabolomics is the study of the metabolites present in a specific sample and therefore, it allows us to evaluate the functional activity carried out by a bacterial community by directly monitoring the final products of its metabolism. This type of method requires that metabolites, usually isolated from body samples (such as urine, feces, and blood) are estimated using various technologies, such as nuclear magnetic resonance (NMR), or microscopy–mass spectrometry [144]. The result is a series of specific absorption spectra (or peaks) sequences, which derive from the range of metabolites present in the sample. A fundamental limitation of this method is that it can be difficult to determine precisely which bacterial species is producing that metabolite [145]. Additionally, this method may be ineffective due to the presence of DNA derived from dead or inactive species. Furthermore, many metabolites (such as short-chain fatty acids) are rapidly absorbed by the host, which means that production levels cannot be accurately attributed for a given bacterial species. Finally, resolution limits mean that only a small subset of the wide range of metabolites that may be present in a complex sample such as feces can be accurately monitored [146].

Stable isotope labeling (SIP) is a functional method that requires the microbial communities of interest to be incubated on substrates containing stable isotopes, such as 13C, 15N, and 18O. At this point, the species that can grow on the substrate provided will incorporate the isotope markers into their cellular biomass, which can then be examined by identifying the elements that compose it, such as DNA/RNA, or proteins or fatty acids derived from phospholipids, which will all be obviously marked. An important limit is the cost of the equipment [147]. Furthermore, labeling with stable isotopes requires that bacteria grow in the presence of the labeled substrates which, therefore, cannot be incorporated by cells and/or organisms. Therefore, the bacterial communities to be studied must be maintained in artificial laboratory conditions, thus not allowing the obtainment of results capable of fully reflecting the activity carried out by the microbiota in vivo [148,149].

## 6. Biomolecular Mechanisms of Intestinal Dysbiosis

**Figure 1:**
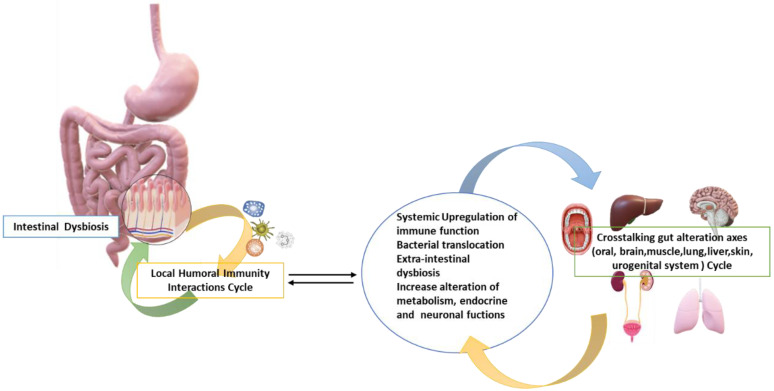
There are complex host–microbe interactions in the gut ranging from direct cell-to-cell to broader systemic communication, involving various organs including the central nervous system (CNS), e.g., diarrhea after broad-spectrum antibiotic treatment for *C. difficile*. Therefore, a cause that leads to an alteration of the qualitative and quantitative composition also leads to a continuous cycle of disharmony (among the local bacterial populations and mucosa), thus creating a state of inflammation in the intestinal mucosa that, if it persists, causes pathogenic bacteria to find room to proliferate and subsequently to move, so that we can define it as a local humoral immunity interactions cycle (LHII). This can lead to an immune upregulation with subsequent imbalance (extraintestinal dysbiosis) of all the microbiota’s axes interconnected with the intestinal microbiota in various organs and, if it persists, it creates a cross-talking gut axes alteration cycle (CGAAC), which leads to an increase in local and systemic dysfunction in the organism’s host over time, creating “reflex” diseases. Credits: Original figure by I.A. Charitos.

The intestinal microbiota and the host coexist in harmony (eubiosis) and from this there is mutual benefit. The host provides the space and suitable conditions (nutrients, presence of O_2_ or microaerophilia, temperature, and pH) for the growth of the microbiota, thus participating in the metabolic pathways of the host, producing useful substances that cannot be produced by the host, or inducing the immune response of the host to various infections. Therefore, the metabolism and fermentation of many nondigestible food components, such as fibers, some lipids and proteins, bile acids, cholesterol, etc., is one of the most important functions of the microbiota in the large intestine (7–10% of the host’s daily energy requirement) [150,151]. In this way the bacteria provide energy but also produce short-chain fatty acids (butyric acid and propionic acid), which are an additional source of energy for the host. These acids have the utility of: (a) supplying energy to colon cells and bacteria, (b) activating the mechanisms that promote the integrity of the tissues of the area, (c) influencing the immune system and immunization, (d) influencing the onset of metabolic diseases (obesity, osteoarthritis, and diabetes diabetes), (e) having anti-inflammatory action, (f) having anti-apoptotic action, (g) regulating lipogenesis, (h) regulating appetite hormones and pH, and (i) contributing to nutrient absorption. Some bacterial species can synthesize amino acids and vitamins (such as K, B12, folic acid, thiamine, biotin, etc.). The *Bacillus thetaiotaomicron* is responsible for the breakdown of polysaccharides that become indigestible in the large intestine through the presence of various enzymes such as glycosides hydrolases and lyases of polysaccharides that break down pectin, arabinose, etc. The “friendly” bacterial species, such as *Lactobacillus* spp. and *Bifidobacteria* spp., lack the proinflammatory external lipopolysaccharide (LPS) chains, which are anchored to the cell walls of pathogenic bacteria such as *E. coli* and the genus *Salmonella* [6,21,152,153,154,155]. Symbiotic bacteria of the microbiota secrete antimicrobials such as bacteriocins and hydrogen peroxide, thus inhibiting the growth of other pathogenic bacteria. There is also competition for both the location of each other, and the availability of present nutrients in the lumen. The microbiota regulate the development and function of the innate and acquired immune systems. In the circumstances of eubiosis, constant stimulation of the immune system by the gut microbiota leads to a state of “low normal inflammation”, which is a direct and effective defense mechanism against pathogens. Furthermore, the flora competes with its protective role, metabolizing the nutrients necessary for the survival of pathogens and producing molecules that inhibit the growth of these bacteria [6,21,156]. Therefore, the function of the intestinal microbiota in terms of the defense of the organism is, on the one hand, to influence the intestinal immune mechanism and, on the other hand, to prevent the possible invasion of pathogens by directly affecting them and/or by “activating” the immune system of the host [157]. In fact, through natural immunity, the molecular patterns associated with characteristic pathogens (PAMP) are identified on the microorganisms, thus selecting potentially pathogenic from nonpathogenic microbes. More specifically, natural immune cells have specific PRRs (pattern recognition receptors) that bind to PAMPs. PRRs are involved in the activation of acquired immunity and the release of cytokines, for example, the Toll-like receptors (TLRs), which are found in macrophages, neutrophils, dendritic cells, and the epithelial cells of the intestinal mucus [158]. PAMPs recognized by PRRs are bacterial carbohydrates (such as lipopolysaccharide-LPS and mannose), nucleic acids (viral DNA or RNA), bacterial peptides (such as flagellin), peptidoglycans, and fungal glucans from liposuction. However, since all of these are present and are also found in symbiotic microbes, they are referred to by the term MAMP (molecular models associated with microbes) [158,159]. Through the recognition of MAMPs, symbiotic microbes change the expression of TLRs in natural immunity cells and trigger the activation of the NF-κβ pathway which stimulates the production of cytokines and ultimately results in the activation of T lymphocytes, i.e., acquired immunity. As we have highlighted, gut germs can change the quantity of mucus produced by the cells of the intestinal mucosa and thus play a protective role in conditions of eubiosis which will activate the body’s defenses, protecting it from pathogens [6,160]. Hence, commensal bacteria prove necessary in eubiosis for the aid of regular digestion, for the normal development/function of the immune system (intestinal, mucosal, and systemic), to lower the pH with short-chain fatty acids (SCFAs), to secrete antitoxin proteins (bacteriocins) against toxin-producing bacteria, and to exert an important defense against colonization by non-commensal microorganisms with the regulation of intestinal mucus. Therefore, there are host–microbe local interactions involving various organs, creating the gut axes (Figure 1) [5,6,7,161,162,163,164].

Thus, under suitable conditions, a long-term symbiosis with many benefits for the host’s health may exist. However, when for some reason the conditions change, the composition of the microbiota also changes, resulting in pathological conditions, infections, inflammations, and various psychosomatic diseases. This condition is called dysbiosis. A dysbiotic state allows the settlement of non-friendly, and therefore pathogenic, bacteria in place of the resident “friendly” commensal bacteria (Table 2) [165,166,167,168,169,170,171,172,173].

The environmental factors mentioned previously, specifically unhealthy lifestyle choices (such as low or exhausting physical activity levels, psychogenic stress, or smoking), exposure to toxic substances (such as industrial chemicals, heavy metals, or abuse of antibiotics), and “bad” diets (overconsumption of sugar, alcohol, caffeine, or spicy foods, low-fiber diet, etc.), activate in combination with the genetic predisposition of the host (idiosyncrasy) to become an abnormal irregulate function of the host’s immune system. This condition can be cause a chronic inflammation of the intestinal mucosa, which in turn is a potential risk factor for idiopathic inflammatory bowel disease (IBD) and other severe chronic diseases [174,175,176,177]. In pathological conditions of the host, such as in the case of Crohn’s disease, the disease is mainly associated with cytokines of T1 helper cells (factor TNF-α, interleukin-12: IL-12, and interferon-γ or IFN-γ). When mucosal injury occurs, epithelial cells are transferred to the site of mucosal injury for healing and rehabilitation. According to recent scientific data, an unexpected immune response to acute injury in Crohn’s disease patients is indicated. People suffering from this IBD show low neutrophil accumulation and lower IL-8 and IL-1β production. There is also talk of damage (defect) in the immunoregulation, which implies the perpetuation (worsening) of inflammation. Crohn’s disease, as in ulcerative colitis, also activates CD4 helper T cells which are responsible for the secretion of proinflammatory cytokines. In contrast with the morbid condition of the host where their activation is observed, CD4 helper T cells and epithelial cells in normal state activate CD8+ suppressor cells [6]. Patients perceive endurance in T-cell apoptosis which is attributed to IL-6. The macrophages and monocytes may release sIL-6R (a soluble interfering receptor) that binds to IL-6, pushes gp130 to the cell surface, and induces anti-apoptotic gene expressions. Even the cells of the mucosa of patients tend to be associated with leukocytes compared to healthy individuals, which indicates that the non-involved cells in the immune response take part in the pro-inflammatory formation of chronic inflammation [174,178,179]. 

Genetic factors are thought to have a direct effect on the composition of the gut microbiota leading to the condition of dysbiosis. The epithelial cells of the intestinal mucosa are the first line of defense against pathogenic microbes. Although these cells are in constant relationships with germs and their products (despite being pro-inflammatory agents for other cell types), they do not react with a defense response. Hence these cells in the intestinal environment provide protection to the host from an inflammatory response against the microbiota. Therefore, the role of intestinal cells is the ability to recognize pathogens [6,179] and only infection with these pathogens will induce a proinflammatory response. It has been found that the NOD2/CARD15 gene participates in this intracellular discrimination system of intestinal epithelial cells. It is also characterized as a cytoplasmic protein, the expression of which is limited to monocytes/macrophages. Furthermore, it can be expressed in other cell types or caused after treatment with proinflammatory agents (IFNγ or TNFα) [179,180]. Its role is attributed to the activation of the NFκβ transcription factor pathway, the main regulator of proinflammatory cytokines (TNFα and IL1b) that induce inflammation. 

Mutations in the NOD2/CARD15 gene inhibit the pathogenic or nonpathogenic microbial identification mechanism, disrupting the normal cytokine inhibition mechanism with consequent dysbiosis of the microbiota leading to significant inflammation of the intestinal mucosa [181,182]. Gut microbiota dysbiosis was noted in mice in which the NOD2/CARD15 gene is not expressed. Indeed, levels of the phyla *Bacteroidetes* and *Bacillota* (such as *Bacilli* spp.), were particularly high in mice that had mutations in this gene versus those that did not. Furthermore, after colonization of the intestine of mice with *Helicobacter hepaticus* the fecal microbiota in the following days of those without mutations showed a greater ability to eliminate this pathogen bacterium; in contract to those that had mutations. Similarly, it also occurs with *H. pylori* for its mutagenic and carcinogenic power in the gastric mucosa [183,184,185,186]. The NOD2 gene contributes to the identification of microorganisms with a harmful effect on the intestinal mucosa, providing host protection from their colonization. It was observed that patients with Crohn’s disease or ulcerative colitis and mutations in the NOD2 gene showed low populations in intestinal biopsies of the genera *Clostridium* XIVa and IV with high presence of *Actinomycetota* and *Pseudomonadota* phyla [187]. It was also noted that individuals with IBD and NOD2 gene mutation present a dysbiosis of the intestinal microbiota with a balanced/disturbed immune system with a high presence of *Enterobacteriaceae* [188]. The ATG16L1 gene regulates the breakdown of proteins in the lysosome, the production of cytokines, and cell homeostasis. A correlation of its mutations with intestinal dysbiosis was observed. Indeed, in individuals with Crohn’s disease (in which the disease was in recession) who had a mutation in the ATG16L1 gene, intense activity of the GRP78 and peIF2α markers was noted. These markers detect the endoplasmic stress of the Paneth cellular network [186]. It has also been noted that if there is an important stress condition, individuals are more likely to develop idiopathic inflammatory disease in the small intestine and may have surgical complications, such as Crohn’s fistulas [189]. Additionally, increased stress indices have shown elevated levels of *Escherichia coli* in intestinal biopsies. Finally, high concentrations of the species *Bacteroides*, *Fusobacteria*, and *E. coli* with low presence of *Lachnospiraceae* (family of bacteria belonging to the order *Clostridiales*) in tissues with inflammation, were observed in patients with IBD with a defect of this gene. An important factor in intestinal microbiota dysbiosis is bacterial translocation, defined as the transport of germs through the intestinal mucosa to sterile areas (mesenteric lymph nodes and abdominal organs) [190,191]. This translocation is observed in patients with Crohn’s disease and ulcerative colitis. Bacterial translocation therefore includes transport through the vulnerable intestinal mucosa of antigens and endotoxins into the systemic circulation, thus inducing the formation of inflammation and damage to various organs. In host conditions, such as inflammatory bowel disease, a hostile environment is formed in the gut with a modified microflora composition; bacterial translocation in these diseases is attributed to either lesions observed in the gut mucosa or mutations in the CARD15 and ATG16L1 genes [190,191,192,193,194]. 

## 7. The Importance of Gut Microbiota Testing to Reveal Host’s Dysbiosis 

As noted, the qualitative (type of bacteria) and quantitative (other changes in the number of species) variations of the gut microbiota relate to the state of well-being of our organism. Unveiling its dysbiotic composition allows us in advance or in time to preserve it or correct it to reach eubiosis. Thus, we can avoid, cure, or reduce the risk of some pathologies, as mentioned. With the presence of advanced molecular techniques for highly sophisticated analyses, it is possible to characterize the components and microbial functionality of the intestinal microbiota with more precision. The analysis of the intestinal microbiota is performed using a special kit for taking a fecal sample (it can be stored for up to 4 weeks at room temperature) (Table 3). The genetic patrimony expressed from the intestinal microbiota, results to be more rich respect of other individual niche, and therefore is indispensable in the homeostasis of overall health. Thus, it was necessary to investigate the composition of the intestinal microbiota, to check their state of well-being; it could therefore, in cases of dysbiosis, indicate a targeted therapy [195,196]. 

It is possible to analyze different parameters even if the aspects most considered and analyzed are the biodiversity index (alpha diversity) and the possible degree of dysbiosis on the composition of the microbiota (a eubiotic microbiota is characterized by a high level of taxonomic diversification). In particular, from the sample, it is possible to obtain: (a) descriptive analysis of the relative abundance of the various bacterial species, (b) the degree of metabolic efficiency, (c) an evaluation of the presence of potentially pathogenic bacterial groups (such as *C. difficile*, *C. perfringens*, *Salmonella*, *Klebsiella*, *Enterococcus faecalis*, etc.), and (d) an evaluation of the physiological functions expressed in “indices” calculated on the basis of the relative overpopulation of the species involved in that function (Table 4) [197]. 

The sample is then analyzed through massive sequencing (next generation sequencing) so it is obtained through a bio-computing processing and statistical analysis of the data for the identification of all the bacterial components of the microbiota in question. Then, the analysis is performed from a sample of 1–2 g of feces, from which the DNA of the bacteria is extracted in the laboratory and then purified and amplified by NGS. Therefore, based on the quantitative and qualitative variations obtained from the sample, a complete and usable picture of how this can impact the physiology of the host is returned by applying a method of functional interpretation [198]. Then the examination of the microbiota detects the “fingerprint” of our bacterial component and analyses its overall state of balance and functionality. Based on the results obtained, it will in fact be possible, if necessary, to adopt the right corrective strategies, such as changes to nutrition or lifestyle, integration with probiotics and/or prebiotics, etc. As mentioned, the test is designed to utilize the rRNA 16S gene as target and amplification primers for PCR and probe for hydrolysis, which enhances the specificity of the dose. Each qPCR DNA microbial DNA sampler analyzes two samples simultaneously [197,199]. The qPCR microbial DNA metabolic distillation matrix is a search tool used for screening or regulating profiling and test strips of test samples, associates, and obesity, type 2 diabetes mellitus, metabolic syndrome, and other diseases. Identification is the determination of the presence of microbes in the sample that enable the excision of a control of the model during analysis [200]. Positive indices that are obtained are important for maintaining the health of the host, so they are obtained when the intestinal microbiota has the characteristics necessary to efficiently perform the indicated function. Instead, the negative indices show a potential of the intestinal microbiota to contribute to the establishment or consolidation of important groups of local or systemic diseases. However, the high values of the indices that are negative by themselves do not represent a diagnosis for certain pathologies because they are obtained when the intestinal microbiota has characteristics and that, in the presence of other predisposing conditions (genetics, environment, comorbidities, lifestyle, and food habits), could represent a further predisposing factor towards the group of pathologies indicated [197,201]. 

## 8. Microbiota, Dysbiosis Disease, and Personalized Management

After an evaluation of the condition of the microbiota as mentioned above, we can manage the patient or the person in a more specific way. With this type of detection of a person’s microbiota we can characterize his dysbiosis and intervene with a targeted therapeutic plan. In fact we must have in mind that the dysbiosis can be: (a) deficiency, resulting from a deficit of the bacterial communities of the intestinal microbiota (*Bifidobacteria* spp. and *Lactobacillus* spp.), mostly favored by a diet poor in soluble fiber and/or rich in packaged, refined, sterilized foods, or consequent to treatments with antibiotics, (b) putrefactive, which is favored by a diet excessively rich in animal fats and meats, and low in fiber with an increase in bacterial populations of *Bacteroides* spp., *Clostridium* spp., *Peptococcus* spp., and *Eubacteria* spp., (c) fermentative, which is characterized by a condition of relative intolerance to carbohydrates or excessive consumption of simple sugars with an increase in bacterial fermentation, (d) sensitization caused by an immune response to components of the normal intestinal microbiota due to deficiency of the immune barrier composed of secretory IgA, and (e) from overgrowing fungi (such as *Candida albicans* and *Saccharomycetes*) favored by a diet rich in simple sugars, leavened foods, refined carbohydrates, and low in fiber [6,21,202]. The various ways to manage and modulate the dysbiotic intestinal microbiota can be dietary interventions (which also include the use of prebiotics, prebiotics, and postbiotics) and fecal transplantation, to mitigate or treat diseases, such as *C. difficile* infection. Therefore, IBD is one of the best studied conditions associated with dysbiosis; it is heterogeneous with three main subtypes: ulcerative colitis, Crohn’s disease, and colitis indeterminate with the microbiome [203,204,205]. These heterogenicities are faced with different therapeutic approaches and therefore the intestinal microbial community present is carefully evaluated. Furthermore, specific diets limiting fermentable oligosaccharides, di-mono-saccharides, and polyols have shown to be beneficial in patients with IBS. In an obese or overweight person, in a metabolic syndrome, or in a patient with type II diabetes, nutritional plans aimed at controlling body weight and restoring the host’s energy metabolism, such as the glycemia balance, can be integrated [206,207].

In cardiovascular diseases and cholesterol metabolism, nutritional plans can be integrated (also with probiotics such as Lactobacillus acidophilus and/or Bifidobacterium Bifidum) and changes to lifestyle introduced (tobacco abuse, consumption of alcohol, and others) to help control them; e.g., trimethylamine oxidase (TMAO) in atherosclerosis and the inhibition of the microbial enzymes trimethylamine lyases (CutC/D and CntA/B) generating trimethylamine (TMA) from various dietary TMA-containing nutrients. The two TMA lyases have been show to restrict substrate specificity for cleaving choline and carnitine, respectively [205,206,208]. The inhibition of TMA lyases can occur by 3, 3-dimethyl-1-butanol (a structural analog of choline) decreasing bacterial TMA production in a high-choline diet-fed murine model and can be found in olive oil, red wine, and other foods [205,206,209]. Finally, the beneficial effects of food interventions with probiotics with a dysbiotic microbiota on anxiety disorders are further evidence of the involvement and influence of the microbiota and on their appearance. Probiotics, such as *L. rhamnosus*, reduced the anxiety of people who exhibited depressive behaviors. The *B. longum* probiotic has a similar effect, while consuming probiotic milk for 3 weeks significantly improved the psychological situation of the people who received it. These probiotic bacterial strains with specific action in affecting the gut–brain axis can be called “psychobiotics”. Furthermore, high doses of prebiotics like trans-galactooligosaccharide (GOS) had a beneficial effect on people with depression [210,211,212].

## 9. Conclusions 

The analysis of the intestinal microbiota highlights potentially pathogenic bacterial groups that are sometimes present in the microbiota even in very low abundances and which can thus take advantage of any alterations in the microbiota to proliferate excessively and cause clinically relevant disorders. Thanks to a particular genetic analysis technology (next generation sequencing, NGS), the gut microbiota test allows us to read the sequence of the genes of the entire microbial community and then categorize them according to the families present in our intestine. 

The presence in high abundance of some of these bacteria has also been associated with various disorders, such as colitis or recurrent diarrhea. The alteration can be caused by both a low and an excessive presence of one or more bacterial groups. Thus, the analysis of the gut microbiota allows us to highlight the following parameters: the biodiversity index (high healthy microbiota index) and the dysbiosis index which measures the degree of alteration of the intestinal microbial ecosystem compared to what is considered a healthy profile. Consequently, we can set up an adequate therapy to modulate towards eubiosis in the intestinal microbiota. 

## Figures and Tables

**Table 1:** The intestinal microbiota’s variations in composition by host’s diet.

Nutritional Habits and Intestinal Microbiota Change	
**Vegan/vegetarian**	*Prevotella* ↑ *Bifidobacteria* ↑ *Faecalibacterium* ↑ *Enterobacteria* ↓ *Pseudomonadota* ↓ *Bacteroides* ↓
**High in saturated fat, sugar, and animal protein, and low in fiber**	*Bacteroides* ↑ *Pseudomonadota* ↑ *Enterobacteria* ↑ *Bifidobacteria* ↓ *Lactobacilli* ↓ *Eubacteria* ↓
**High in monounsaturated or polyunsaturated fats, fiber, and complex carbohydrates, and low in saturated fat**	*Prevotella* ↑ *Bifidobacteria* ↑ *Lactobacilli* ↑ *Eubacteria* ↑ *Enterobacteria* ↓ *Pseudomonadota* ↓
**Gluten-free diet**	*Enterobacteria* ↑ *Roseburia* ↑ *Bifidobacteria* ↓ *Lactobacilli* ↓ *Eubacteria* ↓ *Prevotella* ↓

**Table 2:** The quantitative, qualitative, or functional disorders of the gastrointestinal microbiota in relation to disease.

Gut Microbiota Dysbiosis Bacterial Changes								
Celiac Disease	Anorexia	Allergies	Diabetes Type II	Autism	Obesity	Idiopathic Inflammatory Bowel Disease	Gastric Cancer	Colorectal Cancer
*Bacteroides vulgatus* ↑ *Escherichia coli* ↓ *Clostridium coccoides* ↓	*Methanobrevibacter smithii* ↑	*Lactobacillus* spp. ↓ *Bifidobacterium adolescentis* ↓ *Clostridioides difficile* ↓ *Helicobacter pylori* ↓	*Bacillota* ↓ *Clostridia* ↓ *Bacteroides* and *Prevotella* ↑ versus *Clostridia coccoides*/*Eubacterium rectale* ↓ *Betaproteobacteria* ↑ *Bacteroidota*/*Bacillota* ↑	*Bacteroidota* ↑ *Pseudomonadota* ↑ *Actinomycetota* ↓ *Bacillota* ↓	*Bacteroidota* ↓ *Lactobacillus* ↑ *Bacillota*/*Bacteroidota* ↓ *Methanobrevibacter smithii* ↓	*Bacteroidota* ↓ *Lachnospiraceae* ↓ *Actinomycetota* ↑ *Pseudomonadota* ↑ *Clostridium leptum* ↓ *Clostridium coccoides* ↓ *Faecalibacterium prausnitzii* ↓ *Bacillota*/*Bacteroidota* ↓ *Bifidobacteria* ↓	*Helicobacter pylori* ↑	*Fusobacterium nucleatum* ↑

**Table 3:** Some conditions for which the gut microbiota test might be performed.

Conditions	Action
Obesity or overweight, metabolic syndrome, diabetes type II	Integrate nutritional plans aimed at controlling body weight and restore the host’s energy metabolism balance
Childhood	Promote proper bacterial maturation for young children
Elderly	Limit the effects of aging through immune depression and the onset of inflammatory processes
Pregnancy and breastfeeding	Support the microbial development of the newborn
Early stages of menopause	Better management of metabolic and hormonal change
Presence and/or persistence of intestinal symptoms (including urogenital) of mild or moderate entity	Prevent the course in some possible pathologies
Specific nutritional needs	e.g., an intense athletic activity or at a competitive level to improve performance
Cardiovascular diseases and cholesterol metabolism	Integrate nutritional plans and change the lifestyle (tobacco, alcohol, drugs abuse, and other), aimed at controlling cholesterol, hypertension, and heart attack

**Table 4:** The different parameters and aspects of dysbiosis degree and the physiological functions indices by test research on intestinal microbiota’s host.

Test Analysis of Microbiota’s Actions	
**Activity and Metabolites (Degree of Metabolic Efficiency)**	**Physiological Functions**
*Proteolytic* (allows the degradation of animal proteins; its hyper-expression compromises energy metabolism through the production of bioactive compounds)	Immunomodulation (indicates the ability of the microbiota to properly support the immune system based on the expression of immunomodulating bacteria)
*Mucolytic* (is important to ensure the renewal of the mucosal layer but which, if excessively stimulated, can also lead to damage to the mucosa itself)	Regulation of cognitive and emotional activities (considering the now proven existence of the gut–brain axis, the presence of bacteria capable of producing metabolites that can regulate the state of stress, anxiety, and depression, such as serotonin, is assessed)
*Short-chain fatty acids or SCFAs* (propionate, butyrate, acetate)(is important for the proper functioning of the immune system, energy metabolism, and the integrity of the intestinal barrier)	Resistance to pathogens (also called barrier effect, it expresses the degree to which the microbiota can repress or hinder the colonization and proliferation of potentially harmful microorganisms)
*Lactate* (controls the pH of the intestinal lumen)	Investigate how the possible concentration of predisposing bacterial strains that can contribute to several diseases (such as inflammatory bowel syndrome, metabolic or cardiovascular disorders, or diseases related to aging, and others) can be useful for prevention.
*Hydrogen sulphide* (as its excessive production promotes inflammation and intestinal permeability with consequent bacterial spread in the systemic circulation)	Bacteria influence on the intestinal permeability (some bacteria through the production of specific metabolites bring benefits to the intestinal epithelium, while others undermine its integrity and therefore correct functionality with possible systemic consequences)
*Bacterial lipopolysaccharide* (an endotoxin important for the proper stimulation of the immune system but which if produced in high quantities can lead to various diseases, including autoimmune diseases)	

## Data Availability

Data are contained within the article.

## References

[1] Peterson J., Garges S., Giovanni M., McInnes P., Wang L., Schloss J.A., Bonazzi V., McEwen J.E., Wetterstrand K.A., NIH HMP Working Group (2009). The NIH Human Microbiome Project. Genome Res..

[2] Ursell L.K., Metcalf J.L., Parfrey L.W., Knight R. (2012). Defining the human microbiome. Nutr. Rev..

[3] Thursby E., Juge N. (2017). Introduction to the human gut microbiota. Biochem J..

[4] Kho Z.Y., Lal S.K. (2018). The Human Gut Microbiome—A Potential Controller of Wellness and Disease. Front. Microbiol..

[5] Santacroce L., Charitos I.A., Ballini A., Inchingolo F., Luperto P., De Nitto E., Topi S. (2020). The Human Respiratory System and its Microbiome at a Glimpse. Biology.

[6] Santacroce L., Man A., Charitos I.A., Haxhirexha K., Topi S. (2021). Current knowledge about the connection between health status and gut microbiota from birth to elderly. A narrative review. Front. BioSci. (Landmark Ed.).

[7] Inchingolo A.D., Cazzolla A.P., Di Cosola M., Greco Lucchina A., Santacroce L., Charitos I.A., Topi S., Malcangi G., Hazballa D., Scarano A. (2021). The integumentary system and its microbiota between health and disease. J. Biol. Regul. Homeost. Agents.

[8] Clarridge J.E. (2004). Impact of 16S rRNA gene sequence analysis for identification of bacteria on clinical microbiology and infectious diseases. Clin. Microbiol. Rev..

[9] Oren A., Garrity G.M. (2021). Valid publication of the names of forty-two phyla of prokaryotes. Int. J. Syst. Evol. Microbiol..

[10] Santacroce L., Mavaddati S., Hamedi J., Zeinali B., Ballini A., Bilancia M. (2020). Expressive analysis of gut microbiota in pre- and post-solid organ transplantation using bayesian topic models. Comput. Sci. Its Appl. ICCSA.

[11] Chakravorty S., Helb D., Burday M., Connell N., Alland D. (2007). A detailed analysis of 16S ribosomal RNA gene segments for the diagnosis of pathogenic bacteria. J. Microbiol. Methods.

[12] Yang B., Wang Y., Qian P.Y. (2016). Sensitivity and correlation of hypervariable regions in 16S rRNA genes in phylogenetic analysis. BMC Bioinform..

[13] Cani P.D., Moens de Hase E., Van Hul M. (2021). Gut Microbiota and Host Metabolism: From Proof of Concept to Therapeutic Intervention. Microorganisms.

[14] Rinninella E., Raoul P., Cintoni M., Franceschi F., Miggiano G.A.D., Gasbarrini A., Mele M.C. (2019). What is the Healthy Gut Microbiota Composition? A Changing Ecosystem across Age, Environment, Diet, and Diseases. Microorganisms.

[15] Hattori M., Taylor T.D. (2009). The human intestinal microbiome: A new frontier of human biology. DNA Res..

[16] Stinson L.F., Boyce M.C., Payne M.S., Keelan J.A. (2019). The Not-so-Sterile Womb: Evidence That the Human Fetus Is Exposed to Bacteria Prior to Birth. Front. Microbiol..

[17] Aagaard K., Ma J., Antony K.M., Ganu R., Petrosino J., Versalovic J. (2014). The placenta harbors a unique microbiome. Sci. Transl. Med..

[18] Clemente J.C., Ursell L.K., Parfrey L.W., Knight R. (2012). The impact of the gut microbiota on human health: An integrative view. Cell.

[19] Nuriel-Ohayon M., Neuman H., Koren O. (2016). Microbial Changes during Pregnancy, Birth, and Infancy. Front. Microbiol..

[20] Ardissone A.N., de la Cruz D.M., Davis-Richardson A.G., Rechcigl K.T., Li N., Drew J.C., Murgas-Torrazza R., Sharma R., Hudak M.L., Triplett E.W. (2014). Meconium microbiome analysis identifies bacteria correlated with premature birth. PLoS ONE.

[21] Bottalico L., Castellaneta F., Charitos I.A. (2020). From Hydrotherapy to the Discovery of the Gut Microbiota: The Historical Gastrointestinal Health Concept. Pharmacophore.

[22] Milani C., Duranti S., Bottacini F., Casey E., Turroni F., Mahony J., Belzer C., Delgado Palacio S., Arboleya Montes S., Mancabelli L. (2017). The First Microbial Colonizers of the Human Gut: Composition, Activities, and Health Implications of the Infant Gut Microbiota. Microbiol. Mol. Biol Rev..

[23] Ballini A., Scacco S., Boccellino M., Santacroce L., Arrigoni R. (2020). Microbiota and obesity: Where are we now?. Biology.

[24] Palmer C., Bik E.M., Di Giulio D.B., Relman D.A., Brown P.O. (2007). Development of the human infant intestinal microbiota. PLoS Biol..

[25] Dominguez-Bello M.G., Costello E.K., Contreras M., Magris M., Hidalgo G., Fierer N., Knight R. (2010). Delivery mode shapes the acquisition and structure of the initial microbiota across multiple body habitats in newborns. Proc. Natl. Acad. Sci. USA.

[26] Rodríguez J.M., Murphy K., Stanton C., Ross R.P., Kober O.I., Juge N., Avershina E., Rudi K., Narbad A., Jenmalm M.C. (2015). The composition of the gut microbiota throughout life, with an emphasis on early life. Microb. Ecol. Health Dis..

[27] Biagi E., Quercia S., Aceti A., Beghetti I., Rampelli S., Turroni S., Faldella G., Candela M., Brigidi P., Corvaglia L. (2017). The Bacterial Ecosystem of Mother’s Milk and Infant’s Mouth and Gut. Front. Microbiol..

[28] Topi S., Santacroce L., Bottalico L., Ballini A., Inchingolo A.D., Dipalma G., Charitos I.A., Inchingolo F. (2020). Gastric cancer in history: A perspective interdisciplinary study. Cancers.

[29] Marcobal A., Barboza M., Froehlich J.W., Block D.E., German J.B., Lebrilla C.B., Mills D.A. (2010). Consumption of human milk oligosaccharides by gut-related microbes. J. Agric. Food Chem..

[30] Yoo J.Y., Groer M., Dutra S.V.O., Sarkar A., McSkimming D.I. (2020). Gut Microbiota and Immune System Interactions. Microorganisms.

[31] Nash M.J., Frank D.N., Friedman J.E. (2017). Early Microbes Modify Immune System Development and Metabolic Homeostasis-The Restaurant Hypothesis Revisited. Front. Endocrinol..

[32] Ouwehand A., Isolauri E., Salminen S. (2002). The role of the intestinal microflora for the development of the immune system in early childhood. Eur. J. Nutr..

[33] Walker A.W., Ince J., Duncan S.H., Webster L.M. (2011). Dominant and diet-responsive groups of bacteria within the human colonic microbiota. ISME J..

[34] David L.A., Maurice C.F., Carmody R.N., Gootenberg D.B., Button J.E., Wolfe B.E., Turnbaugh P.J. (2014). Diet rapidly and reproducibly alters the human gut microbiome. Nature.

[35] Tomova A., Bukovsky I., Rembert E., Yonas W., Alwarith J., Barnard N.D., Kahleova H. (2019). The Effects of Vegetarian and Vegan Diets on Gut Microbiota. Front. Nutr..

[36] Wolters M., Ahrens J., Romaní-Pérez M., Watkins C., Sanz Y., Benítez-Páez A., Stanton C., Günther K. (2019). Dietary fat, the gut microbiota, and metabolic health—A systematic review conducted within the MyNewGut project. Clin. Nutr..

[37] Rinninella E., Cintoni M., Raoul P., Lopetuso L.R., Scaldaferri F., Pulcini G., Miggiano G.A.D., Gasbarrini A., Mele M.C. (2019). Food Components and Dietary Habits: Keys for a Healthy Gut Microbiota Composition. Nutrients.

[38] Caio G., Lungaro L., Segata N., Guarino M., Zoli G., Volta U., De Giorgio R. (2020). Effect of Gluten-Free Diet on Gut Microbiota Composition in Patients with Celiac Disease and Non-Celiac Gluten/Wheat Sensitivity. Nutrients.

[39] Gupta V.K., Paul S., Dutta C. (2017). Geography, Ethnicity or Subsistence-Specific Variations in Human Microbiome Composition and Diversity. Front. Microbiol..

[40] Okada H., Kuhn C., Feillet H., Bach J.F. (2010). The ‘hygiene hypothesis’ for autoimmune and allergic diseases: An update. Clin. Exp. Immunol..

[41] Maslowski K.M., Mackay C.R. (2011). Diet, gut microbiota and immune responses. Nat. Immunol..

[42] Muniz L.R., Knosp C., Yeretssian G. (2012). Intestinal antimicrobial peptides during homeostasis, infection, and disease. Front. Immunol..

[43] Pickard J.M., Zeng M.Y., Caruso R., Núñez G. (2017). Gut microbiota: Role in pathogen colonization, immune responses, and inflammatory disease. Immunol. Rev..

[44] Hansson G.C. (2012). Role of mucus layers in gut infection and inflammation. Curr. Opin. Microbiol..

[45] Derrien M., van Passel M.W., van de Bovenkamp J.H., Schipper R.G., de Vos W.M., Dekker J. (2010). Mucin-bacterial interactions in the human oral cavity and digestive tract. Gut Microbes.

[46] Liu H., Shiver A.L., Price M.N., Carlson H.K., Trotter V.V., Chen Y., Escalante V., Ray J., Hern K.E., Petzold C.J. (2021). Functional genetics of human gut commensal Bacteroides thetaiotaomicron reveals metabolic requirements for growth across environments. Cell Rep..

[47] Cornick S., Tawiah A., Chadee K. (2015). Roles and regulation of the mucus barrier in the gut. Tissue Barriers.

[48] Paone P., Cani P.D. (2020). Mucus barrier, mucins and gut microbiota: The expected slimy partners?. Gut.

[49] Migliaccio A., Castoria G., de Falco A., Di Domenico M., Galdiero M., Nola E., Chambon P., Auricchio F. (1991). In vitro phosphorylation and hormone binding activation of the synthetic wild type human estradiol receptor. J. Steroid Biochem. Mol. Biol..

[50] Cockburn D.W., Koropatkin N.M. (2016). Polysaccharide Degradation by the Intestinal Microbiota and Its Influence on Human Health and Disease. J. Mol. Biol..

[51] Chu H., Mazmanian S.K. (2013). Innate immune recognition of the microbiota promotes host-microbial symbiosis. Nat. Immunol..

[52] Ouellette A.J. (2006). Paneth cell alpha-defensin synthesis and function. Curr. Top. Microbiol. Immunol..

[53] Cullen T.W., Schofield W.B., Barry N.A., Putnam E.E., Rundell E.A., Trent M.S., Degnan P.H., Booth C.J., Yu H., Goodman A.L. (2015). Gut microbiota. Antimicrobial peptide resistance mediates resilience of prominent gut commensals during inflammation. Science.

[54] Mukherjee S., Zheng H., Derebe M.G., Callenberg K.M., Partch C.L., Rollins D., Propheter D.C., Rizo J., Grabe M., Jiang Q.X. (2014). Antibacterial membrane attack by a pore-forming intestinal C-type lectin. Nature.

[55] Bien J., Palagani V., Bozko P. (2013). The intestinal microbiota dysbiosis and Clostridium difficile infection: Is there a relationship with inflammatory bowel disease?. Ther. Adv. Gastroenterol..

[56] Macfarlane L.A., Murphy P.R. (2010). MicroRNA: Biogenesis, Function and Role in Cancer. Curr. Genom..

[57] Bi K., Zhang X., Chen W., Diao H. (2020). MicroRNAs Regulate Intestinal Immunity and Gut Microbiota for Gastrointestinal Health: A Comprehensive Review. Genes.

[58] Lu T.X., Rothenberg M.E. (2018). MicroRNA. J. Allergy Clin. Immunol..

[59] Bocchetti M., Ferraro M.G., Ricciardiello F., Ottaiano A., Luce A., Cossu A.M., Scrima M., Leung W.Y., Abate M., Stiuso P. (2021). The Role of microRNAs in Development of Colitis-Associated Colorectal Cancer. Int. J. Mol. Sci..

[60] Ying S.Y., Chang D.C., Lin S.L. (2008). The microRNA (miRNA): Overview of the RNA genes that modulate gene function. Mol. Biotechnol..

[61] Djuranovic S., Nahvi A., Green R. (2012). miRNA-mediated gene silencing by translational repression followed by mRNA deadenylation and decay. Science.

[62] Liu S., da Cunha A.P., Rezende R.M., Cialic R., Wei Z., Bry L., Weiner H.L. (2016). The Host Shapes the Gut Microbiota via Fecal MicroRNA. Cell Host Microbe.

[63] Yoon M.Y., Yoon S.S. (2018). Disruption of the Gut Ecosystem by Antibiotics. Yonsei Med. J..

[64] Jernberg C., Löfmark S., Edlund C., Jansson J.K. (2007). Long-term ecological impacts of antibiotic administration on the human intestinal microbiota. ISME J..

[65] Lewis B.B., Buffie C.G., Carter R.A., Leiner I., Toussaint N.C., Miller L.C. (2015). Loss of Microbiota-Mediated Colonization Resistance to Clostridium difficile Infection with Oral Vancomycin Compared with Metronidazole. J. Infect. Dis..

[66] Eftekharivash L., Hamedi J., Zarrini G., Bakhtiari R. (2021). Acidophilic and Acid Tolerant Actinobacteria as New Sources of Antimicrobial Agents against Helicobacter Pylori. Arch. Razi. Inst..

[67] Stewardson A.J., Gaïa N., François P., Malhotra-Kumar S., Delémont C., Martinez de Tejada B., Schrenzel J., Harbarth S., Lazarevic V. (2015). SATURN WP1 and WP3 Study Groups. Collateral damage from oral ciprofloxacin versus nitrofurantoin in outpatients with urinary tract infections: A culture-free analysis of gut microbiota. Clin. Microbiol. Infect..

[68] Jakobsson H.E., Jernberg C., Andersson A.F., Sjölund-Karlsson M., Jansson J.K., Engstrand L. (2010). Short-term antibiotic treatment has differing long-term impacts on the human throat and gut microbiome. PLoS ONE.

[69] Rineh A., Kelso M.J., Vatansever F., Tegos G.P., Hamblin M.R. (2014). Clostridium difficile infection: Molecular pathogenesis and novel therapeutics. Expert Rev. Anti-Infect. Ther..

[70] Marshall B.M., Levy S.B. (2011). Food animals and antimicrobials: Impacts on human health. Clin. Microbiol. Rev..

[71] Wegener H.C., Aarestrup F.M., Gerner-Smidt P., Bager F. (1999). Transfer of antibiotic resistant bacteria from animals to man. Acta. Vet. Scand. Suppl..

[72] Kristensen N.B., Bryrup T., Allin K.H., Nielsen T., Hansen T.H., Pedersen O. (2016). Alterations in fecal microbiota composition by probiotic supplementation in healthy adults: A systematic review of randomized controlled trials. Genome Med..

[73] Fijan S. (2014). Microorganisms with claimed probiotic properties: An overview of recent literature. Int. J. Environ. Res. Public Health.

[74] Santacroce L., Inchingolo F., Topi S., Del Prete R., Di Cosola M., Charitos I.A., Montagnani M. (2021). Potential beneficial role of probiotics on the outcome of COVID-19 patients: An evolving perspective. Diabetes Metab. Syndr..

[75] Dinleyici E.C., Eren M., Ozen M., Yargic Z.A., Vandenplas Y. (2012). Effectiveness and safety of Saccharomyces boulardii for acute infectious diarrhea. Expert Opin. Biol. Ther..

[76] Servin A.L. (2004). Antagonistic activities of lactobacilli and bifidobacteria against microbial pathogens. FEMS Microbiol. Rev..

[77] Cantore S., Ballini A., De Vito D., Abbinante A., Altini V., Dipalma G., Inchingolo F., Saini R. (2018). Clinical results of improvement in periodontal condition by administration of oral Probiotics. J. Biol. Regul. Homeost. Agents.

[78] Cleusix V., Lacroix C., Vollenweider S., Duboux M., Le Blay G. (2007). Inhibitory activity spectrum of reuterin produced by Lactobacillus reuteri against intestinal bacteria. BMC Microbiol..

[79] Akbari V., Hendijani F. (2016). Effects of probiotic supplementation in patients with type 2 diabetes: Systematic review and meta-analysis. Nutr. Rev..

[80] Wu Y., Zhang Q., Ren Y., Ruan Z. (2017). Effect of probiotic Lactobacillus on lipid profile: A systematic review and meta-analysis of randomized, controlled trials. PLoS ONE.

[81] Borody T.J., Campbell J. (2011). Fecal microbiota transplantation: Current status and future directions. Expert Rev. Gastroenterol. Hepatol..

[82] Signorini L., Ballini A., Arrigoni R., De Leonardis F., Saini R., Cantore S., Inchingolo F. (2021). Evaluation of a Nutraceutical Product with Probiotics, Vitamin D, Plus Banaba Leaf Extracts (*Lagerstroemia speciosa*) in Glycemic Control. Endocr. Metab. Immune Disord. Drug Targets.

[83] Shah A., Macdonald G.A., Morrison M., Holtmann G. (2020). Targeting the Gut Microbiome as a Treatment for Primary Sclerosing Cholangitis: A Conceptional Framework. Am. J. Gastroenterol..

[84] De Vrese M., Marteau P.R. (2007). Probiotics and prebiotics: Effects on diarrhea. J. Nutr..

[85] Parnell J.A., Reimer R.A. (2012). Prebiotic fiber modulation of the gut microbiota improves risk factors for obesity and the metabolic syndrome. Gut Microbes.

[86] Holscher H.D. (2017). Dietary fiber and prebiotics and the gastrointestinal microbiota. Gut Microbes.

[87] Man A., Ciurea C.N., Pasaroiu D., Savin A.I., Toma F., Sular F., Mare A. (2017). New perspectives on the nutritional factors influencing growth rate of *Candida albicans* in diabetics. An in vitro study. Mem. Inst. Oswaldo Cruz.

[88] Arrigoni R., Ballini A., Santacroce L., Cantore S., Inchingolo A., Inchingolo F., Boccellino M. (2022). Another look at dietary polyphenols: Challenges in cancer prevention and treatment. Curr. Med. Chem..

[89] Ling Z., Jin C., Xie T., Cheng Y., Li L., Wu N. (2016). Alterations in the Fecal Microbiota of Patients with HIV-1 Infection: An Observational Study in A Chinese Population. Sci. Rep..

[90] Schroeder B.O., Birchenough G.M.H., Ståhlman M., Arike L., Johansson M.E., Hansson G.C., Bäckhed F. (2018). Bifidobacteria or Fiber Protects against Diet-Induced Microbiota-Mediated Colonic Mucus Deterioration. Cell Host Microbe.

[91] Earle K.A., Billings G., Sigal M., Lichtman J.S., Hansson G.C., Elias J.E., Amieva M., Huang K.C., Sonnenburg J.L. (2015). Quantitative Imaging of Gut Microbiota Spatial Organization. Cell Host Microbe.

[92] Nataraj B.H., Ali S.A., Behare P.V., Yadav H. (2020). Postbiotics-parabiotics: The new horizons in microbial biotherapy and functional foods. Microb. Cell Fact..

[93] Swanson K.S., Gibson G.R., Hutkins R., Reimer R.A., Reid G., Verbeke K., Scott K.P., Holscher H.D., Azad M.B., Delzenne N.M. (2020). The International Scientific Association for Probiotics and Prebiotics (ISAPP) consensus statement on the definition and scope of synbiotics. Nat. Rev. Gastroenterol. Hepatol..

[94] Żółkiewicz J., Marzec A., Ruszczyński M., Feleszko W. (2020). Postbiotics-A Step Beyond Pre- and Probiotics. Nutrients.

[95] Russo E., Giudici F., Fiorindi C., Ficari F., Scaringi S., Amedei A. (2019). Immunomodulating Activity and Therapeutic Effects of Short Chain Fatty Acids and Tryptophan Post-biotics in Inflammatory Bowel Disease. Front. Immunol..

[96] Vallejo-Cordoba B., Castro-López C., García H.S., González-Córdova A.F., Hernández-Mendoza A. (2020). Postbiotics and paraprobiotics: A review of current evidence and emerging trends. Adv. Food Nutr. Res..

[97] Siciliano R.A., Reale A., Mazzeo M.F., Morandi S., Silvetti T., Brasca M. (2021). Paraprobiotics: A New Perspective for Functional Foods and Nutraceuticals. Nutrients.

[98] Gupta S., Allen-Vercoe E., Petrof E.O. (2016). Fecal microbiota transplantation: In perspective. Ther. Adv. Gastroenterol..

[99] Bibbò S., Settanni C.R., Porcari S., Bocchino E., Ianiro G., Cammarota G., Gasbarrini A. (2020). Fecal Microbiota Transplantation: Screening and Selection to Choose the Optimal Donor. J. Clin. Med..

[100] Khoruts A., Sadowsky M.J. (2016). Understanding the mechanisms of faecal microbiota transplantation. Nat. Rev. Gastroenterol. Hepatol..

[101] Ser H.L., Letchumanan V., Goh B.H., Wong S.H., Lee L.H. (2021). The Use of Fecal Microbiome Transplant in Treating Human Diseases: Too Early for Poop?. Front. Microbiol..

[102] Van Nood E., Vrieze A., Nieuwdorp M., Fuentes S., Zoetendal E.G., De Vos W.M., Visser C.E., Kuijper E.J., Bartelsman J.F.W.M., Tijssen J.G.P. (2013). Duodenal infusion of donor feces for recurrent Clostridium difficile. N. Engl. J. Med..

[103] Wilson B.C., Vatanen T., Cutfield W.S., O’Sullivan J.M. (2019). The Super-Donor Phenomenon in Fecal Microbiota Transplantation. Front. Cell. Infect. Microbiol..

[104] Segeritz C.P., Vallier L., Jalali M., Saldanha F.Y.L., Jalali M. (2017). Cell culture: Growing cells as model systems in vitro. Basic Science Methods for Clinical Researchers.

[105] McPherson J.D. (2014). A defining decade in DNA sequencing. Nat. Methods.

[106] Belenguer A., Duncan S.H., Calder A.G., Holtrop G., Louis P., Lobley G.E., Flint H.J. (2006). Two routes of metabolic cross feeding between Bifidobacterium adolescentis and butyrate-producing anaerobes from the human gut. Appl. Environ. Microbiol..

[107] Flint H.J., Duncan S.H., Scott K.P., Louis P. (2007). Interactions and competition within the microbial community of the human colon: Links between diet and health. Environ. Microbiol..

[108] Ferenci T. (1999). ‘Growth of bacterial cultures’ 50 years on: Towards an uncertainty principle instead of constants in bacterial growth kinetics. Res. Microbiol..

[109] Minihane B.J., Brown D.E. (1986). Fed-batch culture technology. Biotechnol. Adv..

[110] Miller T.L., Wolin M.J. (1981). Fermentation by the human large intestine microbial community in an in vitro semi continuous culture system. Appl. Environ. Microbiol..

[111] Winder C.L., Lanthaler K. (2011). The use of continuous culture in systems biology investigations. Methods Enzymol..

[112] Mateles R.I., Battat E. (1974). Continuous culture used for media optimization. Appl. Microbiol..

[113] Kostic A.D., Howitt M.R., Garrett W.S. (2013). Exploring host-microbiota interactions in animal models and humans. Genes Dev..

[114] Turner P.V. (2018). The role of the gut microbiota on animal model reproducibility. Anim. Models Exp. Med..

[115] Ericsson A.C., Davis J.W., Spollen W., Bivens N., Givan S., Hagan C.E., McIntosh M., Franklin C.L. (2015). Effects of vendor and genetic background on the composition of the fecal microbiota of inbred mice. PLoS ONE.

[116] Spath K., Babariya D., Konstantinidis M., Lowndes J., Child T., Grifo J.A., Poulton J., Wells D. (2021). Clinical application of sequencing-based methods for parallel preimplantation genetic testing for mitochondrial DNA disease and aneuploidy. Fertil. Steril..

[117] Valones M.A., Guimarães R.L., Brandão L.A., de Souza P.R., de Albuquerque Tavares Carvalho A., Crovela S. (2009). Principles and applications of polymerase chain reaction in medical diagnostic fields: A review. Braz. J. Microbiol..

[118] Schmidt T.S., Matias Rodrigues J.F., von Mering C. (2014). Ecological consistency of SSU rRNA-based operational taxonomic units at a global scale. PLoS Comput. Biol..

[119] Stuppia L., Antonucci I., Palka G., Gatta V. (2012). Use of the MLPA assay in the molecular diagnosis of gene copy number alterations in human genetic diseases. Int. J. Mol. Sci..

[120] Fraser C.M., Eisen J.A., Nelson K.E., Paulsen I.T., Salzberg S.L. (2002). The value of complete microbial genome sequencing (you get what you pay for). J. Bacteriol..

[121] Sanger F., Nicklen S., Coulson A.R. (1992). DNA sequencing with chain-terminating inhibitors. Biotechnology.

[122] Rouzier C., Chaussenot A., Serre V., Fragaki K., Bannwarth S., Ait-El-Mkadem S., Attarian S., Kaphan E., Cano A., Delmont E. (2014). Quantitative multiplex PCR of short fluorescent fragments for the detection of large intragenic POLG rearrangements in a large French cohort. Eur. J. Hum. Genet..

[123] Thomas T., Gilbert J., Meyer F. (2012). Metagenomics—A guide from sampling to data analysis. Microb. Inform. Exp..

[124] Gill S.R., Pop M., DeBoy R.T., Eckburg P.B., Turnbaugh P.J., Samuel B.S., Gordon J.I., Relman D.A., Fraser-Liggett C.M., Nelson K.E. (2006). Metagenomic analysis of the human distal gut microbiome. Science.

[125] Raghunathan A., Ferguson H.R., Bornarth C.J., Song W., Driscoll M., Lasken R.S. (2005). Genomic DNA amplification from a single bacterium. Appl. Environ. Microbiol..

[126] Marcy Y., Ouverney C., Bik E.M., Lösekann T., Ivanova N., Martin H.G., Szeto E., Platt D., Hugenholtz P., Relman D.A. (2007). Dissecting biological dark matter with single-cell genetic analysis of rare and uncultivated TM7 microbes from the human mouth. Proc. Natl. Acad. Sci. USA.

[127] Zhang Y., Thompson K.N., Branck T., Yan Y., Nguyen L.H., Franzosa E.A., Huttenhower C. (2021). Metatranscriptomics for the Human Microbiome and Microbial Community Functional Profiling. Annu. Rev. Biomed. Data Sci..

[128] Abu-Ali G.S., Mehta R.S., Lloyd-Price J., Mallick H., Branck T., Ivey K.L., Drew D.A., DuLong C., Rimm E., Izard J. (2018). Metatranscriptome of human faecal microbial communities in a cohort of adult men. Nat. Microbiol..

[129] Reck M., Tomasch J., Deng Z., Jarek M., Husemann P., Wagner-Döbler I. (2015). Stool metatranscriptomics: A technical guideline for mRNA stabilization and isolation. BMC Genom..

[130] Gillespie P., Ladame S., O’Hare D. (2018). Molecular methods in electrochemical microRNA detection. Analyst.

[131] Tomasello G., Mazzola M., Jurjus A., Cappello F., Carini F., Damiani P., Gerges Geagea A., Zeenny M.N., Leone A. (2017). The fingerprint of the human gastrointestinal tract microbiota: A hypothesis of molecular mapping. J. Biol. Regul. Homeost. Agents.

[132] Hassibi A., Vikalo H., Riechmann J.L., Hassibi B. (2009). Real-time DNA microarray analysis. Nucleic Acids Res..

[133] Bumgarner R. (2013). Overview of DNA microarrays: Types, applications, and their future. Curr. Protoc. Mol. Biol..

[134] Pettini F., Savino M., Corsalini M., Cantore S., Ballini A. (2015). Cytogenetic genotoxic investigation in peripheral blood lymphocytes of subjects with dental composite restorative filling materials. J. Biol. Regul. Homeost. Agents.

[135] Wong W., Farr R., Joglekar M., Januszewski A., Hardikar A. (2015). Probe-based Real-time PCR Approaches for Quantitative Measurement of microRNAs. J. Vis. Exp..

[136] Dymond J.S. (2013). Explanatory chapter: Quantitative PCR. Methods Enzymol..

[137] Brich S., Bozzi F., Perrone F., Tamborini E., Cabras A.D., Deraco M., Stacchiotti S., Dagrada G.P., Pilotti S. (2020). Fluorescence in situ hybridization (FISH) provides estimates of minute and interstitial BAP1, CDKN2A, and NF2 gene deletions in peritoneal mesothelioma. Mod. Pathol..

[138] Ratan Z.A., Zaman S.B., Mehta V., Haidere M.F., Runa N.J., Akter N. (2017). Application of Fluorescence In Situ Hybridization (FISH) Technique for the Detection of Genetic Aberration in Medical. Sci. Cureus.

[139] Meng Z., Wang Q., Khurshid H., Raza G., Han J., Wang B., Wang K. (2021). Chromosome Painting Provides Insights into the Genome Structure and Evolution of Sugarcane. Front. Plant Sci..

[140] Schiebenhoefer H., Schallert K., Renard B.Y., Trappe K., Schmid E., Benndorf D., Riedel K., Muth T., Fuchs S. (2020). A complete and flexible workflow for metaproteomics data analysis based on MetaProteomeAnalyzer and Prophane. Nat. Protoc..

[141] Feng S., Sterzenbach R., Guo X. (2021). Deep learning for peptide identification from metaproteomics datasets. J. Proteom..

[142] Li S., Tang H., Ye Y. (2019). A Meta-Proteogenomic Approach to Peptide Identification Incorporating Assembly Uncertainty and Genomic Variation. Mol. Cell. Proteom..

[143] Clish C.B. (2015). Metabolomics: An emerging but powerful tool for precision medicine. Cold Spring Harb. Mol. Case Stud..

[144] Sampson J.N., Boca S.M., Shu X.O., Stolzenberg-Solomon R.Z., Matthews C.E., Hsing A.W., Tan Y.T., Ji B.T., Chow W.H., Cai Q. (2013). Metabolomics in epidemiology: Sources of variability in metabolite measurements and implications. Cancer Epidemiol. Biomark. Prev..

[145] Klupczyńska A., Dereziński P., Kokot Z.J. (2015). Metabolomics in medical sciences—Trends; challenges and perspectives. Acta Pol. Pharm..

[146] Jiang B., Jin N., Xing Y., Su Y., Zhang D. (2018). Unraveling uncultivable pesticide degraders via stable isotope probing (SIP). Crit. Rev. Biotechnol..

[147] Wilhelm R., Szeitz A., Klassen T.L., Mohn W.W. (2014). Sensitive, Efficient Quantitation of 13C-Enriched Nucleic Acids via Ultrahigh-Performance Liquid Chromatography-Tandem Mass Spectrometry for Applications in Stable Isotope Probing. Appl. Environ. Microbiol..

[148] Uhlík O., Jecná K., Leigh M.B., Macková M., Macek T. (2009). DNA-based stable isotope probing: A link between community structure and function. Sci. Total Environ..

[149] Tremaroli V., Backhed F. (2012). Functional interactions between the gut microbiota and host metabolism. Nature.

[150] Purchiaroni F., Tortora A., Gabrielli M., Bertucci F., Gigante G., Ianiro G., Ojetti V., Scarpellini E., Gasbarrini A. (2013). The role of intestinal microbiota and the immune system. Eur. Rev. Med. Pharmacol. Sci..

[151] Macfarlane S., Macfarlane G.T. (2003). Regulation of short-chain fatty acid production. Proc. Nutr. Soc..

[152] Mangiola F., Ianiro G., Franceschi F., Fagiuoli S., Gasbarrini G., Gasbarrini A. (2016). Gut microbiota in autism and mood disorders. World J. Gastroenterol..

[153] Srikanth C.V., MCCormick O.R.M.I.C.K.B.A. (2008). Interactions of the intestinal epithelial with the pathogen and the indigenous microbiota: A three-way crosstalk. Interdiscip. Perspect. Infect. Dis..

[154] Nakkarach A., Foo H.L., Song A.A., Nitisinprasert S., Withayagiat U. (2020). Promising discovery of beneficial Escherichia coli in the human gut. 3 Biotech.

[155] Garcia-Gutierrez E., Mayer M.J., Cotter P.D., Narbad A. (2019). Gut microbiota as a source of novel antimicrobials. Gut Microbes.

[156] Kamada N., Chen G.Y., Inohara N., Núñez G. (2013). Control of pathogens and pathobionts by the gut microbiota. Nat. Immunol..

[157] Vance R.E., Isberg R.R., Portnoy D.A. (2009). Patterns of pathogenesis: Discrimination of pathogenic and nonpathogenic microbes by the innate immune system. Cell Host Microbe.

[158] Mogensen T.H. (2009). Pathogen recognition and inflammatory signaling in innate immune defenses. Clin. Microbiol. Rev..

[159] Kumagai Y., Akira S. (2010). Identification and functions of pattern-recognition receptors. J. Allergy Clin. Immunol..

[160] Sun Y., O’Riordan M.X. (2013). Regulation of bacterial pathogenesis by intestinal short-chain Fatty acids. Adv. Appl. Microbiol..

[161] Ray K. (2020). The oral-gut axis in IBD. Nat. Rev. Gastroenterol. Hepatol..

[162] Albillos A., de Gottardi A., Rescigno M. (2020). The gut-liver axis in liver disease: Pathophysiological basis for therapy. J. Hepatol..

[163] Amabebe E., Anumba D.O.C. (2020). Female Gut and Genital Tract Microbiota-Induced Crosstalk and Differential Effects of Short-Chain Fatty Acids on Immune Sequelae. Front. Immunol..

[164] Lovreglio P., Bukvic N., Fustinoni S., Ballini A., Drago I., Foà V., Guanti G., Soleo L. (2006). Lack of genotoxic effect in workers exposed to very low doses of 1,3-butadiene. Arch. Toxicol..

[165] Lathrop S.K., Bloom S.M., Rao S.M., Nutsch K., Lio C.W., Santacruz N., Peterson D.A., Stappenbeck T.S., Hsieh C.S. (2011). Peripheral education of the immune system by colonic commensal microbiota. Nature.

[166] Armougom F., Henry M., Vialettes B., Raccah D., Raoult D. (2009). Monitoring bacterial community of human gut microbiota reveals an increase in Lactobacillus in obese patients and Methanogens in anorexic patients. PLoS ONE.

[167] Round J.L., Lee S.M., Li J., Tran G., Jabri B., Chatila T.A., Mazmanian S.K. (2011). The Toll-like receptor 2 pathway establishes colonization by a commensal of the human microbiota. Science.

[168] Muñoz-Garach A., Diaz-Perdigones C., Tinahones F.J. (2016). Gut microbiota and type 2 diabetes mellitus. Endocrinol. Nutr..

[169] Li Q., Han Y., Dy A.B.C., Hagerman R.J. (2017). The Gut Microbiota and Autism Spectrum Disorders. Front. Cell. Neurosci..

[170] Aoun A., Darwish F., Hamod N. (2020). The Influence of the Gut Microbiome on Obesity in Adults and the Role of Probiotics, Prebiotics, and Synbiotics for Weight Loss. Prev. Nutr. Food Sci..

[171] Kostic A.D., Chun E., Robertson L., Glickman J.N., Gallini C.A., Michaud M., Clancy T.E., Chung D.C., Lochhead P., Hold G.L. (2013). Fusobacterium nucleatum potentiates intestinal tumorigenesis and modulates the tumor-immune microenvironment. Cell Host Microbe.

[172] Polimeno L., Barone M., Mosca A., Viggiani M.T., Di Leo A., Debellis L., Troisi M., Daniele A., Santacroce L. (2019). Gut Microbiota Imbalance is Related to Sporadic Colorectal Neoplasms. A Pilot Study. Appl. Sci..

[173] Francino M.P. (2016). Antibiotics and the human gut microbiome: Dysbioses and accumulation of resistances. Front. Microbiol..

[174] Man A., Mare A., Toma F., Curticăpean A., Santacroce L. (2016). Health Threats from Contamination of Spices Commercialized in Romania: Risks of Fungal and Bacterial Infections. Endocr. Metab. Immune Disord. Drug Targets.

[175] Myles I.A. (2014). Fast food fever: Reviewing the impacts of the Western diet on immunity. Nutr. J..

[176] Polimeno L., Barone M., Mosca A., Viggiani M.T., Joukar F., Mansour-Ghanaei F., Mavaddati S., Daniele A., Debellis L., Bilancia M. (2020). Soy Metabolism by Gut Microbiota from Patients with Precancerous Intestinal Lesions. Microorganisms.

[177] Macfarlane S., Steed H., Macfarlane G.T. (2009). Intestinal bacteria and inflammatory bowel disease. Crit. Rev. Clin. Lab. Sci..

[178] Caruso R., Warner N., Inohara N., Núñez G. (2014). NOD1 and NOD2, signaling, host defense, and inflammatory disease. Immunity.

[179] Philpott D. (2009). Dana Philpott: Exploring the land of NOD. Interview by Kira Heller. J. Exp. Med..

[180] Abraham C., Cho J.H. (2006). Functional consequences of NOD2 (CARD15) mutations. Inflamm. Bowel Dis..

[181] Oeckinghaus A., Ghosh S. (2009). The NF-kappaB family of transcription factors and its regulation. Cold Spring Harb. Perspect. Biol..

[182] Atherly T., Mosher C., Wang C., Hostetter J., Proctor A., Brand M.W., Phillips G.J., Wannemuehler M., Jergens A.E. (2016). Helicobacter bilis Infection Alters Mucosal Bacteria and Modulates Colitis Development in Defined Microbiota Mice. Inflamm. Bowel Dis..

[183] Santacroce L., Cagiano R., Del Prete R., Bottalico L., Sabatini R., Carlaio R.G., Prejbeanu R., Vermesan H., Dragulescu S.I., Vermesan D. (2008). *Helicobacter pylori* infection and gastric MALTomas: An up-to-date and therapy highlight. Clin. Ter..

[184] Santacroce L., Bufo P., Latorre V., Losacco T. (2000). Ruolo dei mastociti nella fisiopatologia delle lesioni gastriche indotte da *Helicobacter pylori* [Role of mast cells in the physiopathology of gastric lesions caused by *Helicobacter pylori*]. Chir. Ital..

[185] Forbes J.D., Van Domselaar G., Bernstein C.N. (2016). The Gut Microbiota in Immune-Mediated Inflammatory Diseases. Front. Microbiol..

[186] Al Nabhani Z., Dietrich G., Hugot J.P., Barreau F. (2017). Nod2: The intestinal gate keeper. PLoS Pathog..

[187] Chen F., Amgalan D., Kitsis R.N., Pessin J.E., Feng D. (2020). ATG16L1 autophagy pathway regulates BAX protein levels and programmed cell death. J. Biol. Chem..

[188] Deuring J.J., Fuhler G.M., Konstantinov S.R., Peppelenbosch M.P., Kuipers E.J., de Haar C., van der Woude C.J. (2014). Genomic ATG16L1 risk allele-restricted Paneth cell ER stress in quiescent Crohn’s disease. Gut.

[189] Rizzo A., Losacco A., Carratelli C.R., Domenico M.D., Bevilacqua N. (2013). Lactobacillus plantarum reduces Streptococcus pyogenes virulence by modulating the IL-17, IL-23 and Toll-like receptor 2/4 expressions in human epithelial cells. Int. Immunopharmacol..

[190] Balzan S., de Almeida Quadros C., de Cleva R., Zilberstein B., Cecconello I. (2007). Bacterial translocation: Overview of mechanisms and clinical impact. J. Gastroenterol. Hepatol..

[191] Deitch E.A. (2002). Bacterial translocation or lymphatic drainage of toxic products from the gut: What is important in human beings?. Surgery.

[192] Vaishnavi C. (2013). Translocation of gut flora and its role in sepsis. Indian J. Med. Microbiol..

[193] Lauriola M., Ugolini G., Rivetti S., Nanì S., Rosati G., Zanotti S., Montroni I., Manaresi A., Zattoni D., Belluzzi A. (2011). IL23R, NOD2/CARD15, ATG16L1 and PHOX2B polymorphisms in a group of patients with Crohn’s disease and correlation with sub-phenotypes. Int. J. Mol. Med..

[194] Polimeno L., Francavilla A., Piscitelli D., Fiore M.G., Polimeno R., Topi S., Haxhirexha K., Ballini A., Daniele A., Santacroce L. (2020). The role of PIAS3, p-STAT3 and ALR in colorectal cancer: New translational molecular features for an old disease. Eur. Rev. Med. Pharmacol. Sci..

[195] Allaband C., McDonald D., Vázquez-Baeza Y., Minich J.J., Tripathi A., Brenner D.A., Loomba R., Smarr L., Sandborn W.J., Schnabl B. (2019). Microbiome 101: Studying, Analyzing, and Interpreting Gut Microbiome Data for Clinicians. Clin. Gastroenterol. Hepatol..

[196] Karstens L., Siddiqui N.Y., Zaza T., Barstad A., Amundsen C.L., Sysoeva T.A. (2021). Benchmarking DNA isolation kits used in analyses of the urinary microbiome. Sci. Rep..

[197] Galloway-Peña J., Hanson B. (2020). Tools for Analysis of the Microbiome. Dig. Dis. Sci..

[198] Tang Q., Jin G., Wang G., Liu T., Liu X., Wang B., Cao H. (2020). Current Sampling Methods for Gut Microbiota: A Call for More Precise Devices. Front. Cell. Infect. Microbiol..

[199] Bharti R., Grimm D.G. (2021). Current challenges and best-practice protocols for microbiome analysis. Brief Bioinform..

[200] Lim M.Y., Park Y.S., Kim J.H., Nam Y.D. (2020). Evaluation of fecal DNA extraction protocols for human gut microbiome studies. BMC Microbiol..

[201] (2021). Atlasbiomed, Microbiomedtest. https://atlasbiomed.com/blog/microbiome-test-results-guide/.

[202] DeGruttola A.K., Low D., Mizoguchi A., Mizoguchi E. (2016). Current Understanding of Dysbiosis in Disease in Human and Animal Models. Inflamm. Bowel Dis..

[203] Singh R., Zogg H., Wei L., Bartlett A., Ghoshal U.C., Rajender S., Ro S. (2021). Gut Microbial Dysbiosis in the Pathogenesis of Gastrointestinal Dysmotility and Metabolic Disorders. J. Neurogastroenterol. Motil..

[204] Honkanen J., Vuorela A., Muthas D., Orivuori L., Luopajärvi K., Tejesvi M.V.G., Lavrinienko A., Pirttilä A.M., Fogarty C.L., Härkönen T. (2020). Fungal Dysbiosis and Intestinal Inflammation in Children with Beta-Cell Autoimmunity. Front. Immunol..

[205] Behrouzi A., Nafari A.H., Siadat S.D. (2019). The significance of microbiome in personalized medicine. Clin. Transl. Med..

[206] Purna C., Kashyap P.C., Chia N., Nelson H., Segal E., Elinav E. (2017). Microbiome at the Frontier of Personalized Medicine. Mayo Clin. Proc..

[207] Biesiekierski J.R., Jalanka J., Staudacher H.M. (2019). Can Gut Microbiota Composition Predict Response to Dietary Treatments?. Nutrients.

[208] Janeiro M.H., Ramírez M.J., Milagro F.I., Martínez J.A., Solas M. (2018). Implication of Trimethylamine N-Oxide (TMAO) in Disease: Potential Biomarker or New Therapeutic Target. Nutrients.

[209] Wang Z., Roberts A.B., Buffa J.A., Levison B.S., Zhu W., Org E., Gu X., Huang Y., Zamanian-Daryoush M., Culley M.K. (2015). Non-lethal Inhibition of Gut Microbial Trimethylamine Production for the Treatment of Atherosclerosis. Cell.

[210] Sarkar A., Lehto S.M., Harty S., Dinan T.G., Cryan J.F., Burnet P.W.J. (2016). Psychobiotics and the Manipulation of Bacteria-Gut-Brain Signals. Trends Neurosci..

[211] Cohen Kadosh K., Basso M., Knytl P., Johnstone N., Lau J.Y.F., Gibson G.R. (2021). Psychobiotic interventions for anxiety in young people: A systematic review and meta-analysis, with youth consultation. Transl. Psychiatry..

[212] Johnstone N., Milesi C., Burn O., van den Bogert B., Nauta A., Hart K., Sowden P., Burnet P.W.J., Cohen Kadosh K. (2021). Anxiolytic effects of a galacto-oligosaccharides prebiotic in healthy females (18–25 years) with corresponding changes in gut bacterial composition. Sci. Rep..

